# Periodontal status and the incidence of selected bacterial pathogens in periodontal pockets and vascular walls in patients with atherosclerosis and abdominal aortic aneurysms

**DOI:** 10.1371/journal.pone.0270177

**Published:** 2022-08-11

**Authors:** Agnieszka Kręgielczak, Barbara Dorocka-Bobkowska, Ryszard Słomski, Grzegorz Oszkinis, Zbigniew Krasiński

**Affiliations:** 1 Department of Gerontology and Oral Pathology, Poznan University of Medical Sciences, Poznan, Poland; 2 Institute of Human Genetics, Polish Academy of Sciences, Warsaw, Poland; 3 Department of Vascular and General Surgery, Institute of Medical Sciences, Opole University, Opole, Poland; 4 Department of Vascular, Endovascular Surgery, Angiology and Phlebology, Poznan University of Medical Sciences, Poznan, Poland; University of Sheffield, UNITED KINGDOM

## Abstract

The aim of the study was to examine the periodontal status of patients with atherosclerosis and abdominal aortic aneurysms. The occurrence of 5 periodontopathogens was evaluated in periodontal pockets and atheromatous plaques together with specimens from pathologically changed vascular walls of aortic aneurysms. The study comprised 39 patients who qualified for vascular surgeries. Patients with periodontitis and concomitant atherosclerosis or aneurysms were enrolled in the study. Periodontal indices were evaluated, and subgingival plaque samples were examined together with atheromatous plaques or specimens from vascular walls to identify, by polymerase chain reaction (PCR), the following periodontopathogens: *Porphyromonas gingivalis*, *Tanarella forsythia*, *Aggregatibacter actinomycetemcomitans*, *Prevotella intermedia* and *Treponema denticola*. The majority of patients had chronic severe generalized periodontitis in stages III and IV. Laboratory investigations showed the occurrence of one or more of the five targeted periodontopathogens in 94.6% of the periodontal pockets examined. Of the examined periodontopathogens, only *Porphyromonas gingivalis* was confirmed in 1 atheromatous plaque sample collected from the wall of an aortic aneurysm. Therefore, the occurrence of this bacterium in these vessels was considered to be occasional in patients with chronic periodontitis.

## Introduction

**Fig 1:**
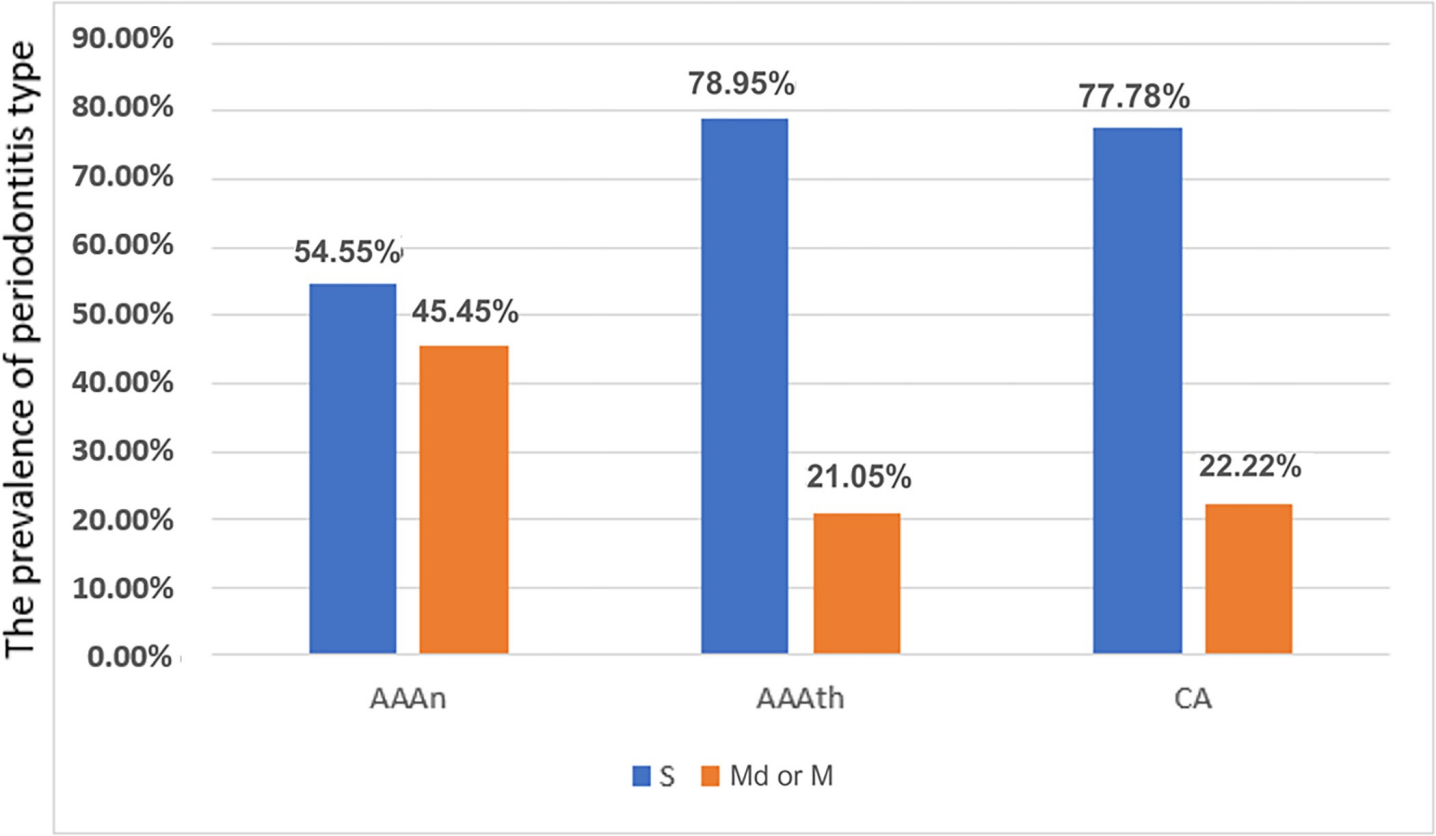
Mechanisms of periodontitis and atherosclerosis correlation.

Chronic periodontitis is a noncommunicable infectious disease resulting in inflammation within the soft and hard tissues supporting teeth, leading to progressive loss of attachment and bone. The condition has a high prevalence affecting 45–50% of the general population, with 38% over age 30 and 69% over age 65 [1]. Current epidemiological data indicate that 41.5% of adults in Poland aged 35–44 years suffer from moderate or chronic periodontitis [2]. According to Tonetti et al. [3], severe periodontitis is the 6^th^ most prevalent disease worldwide. A number of studies have shown that chronic inflammatory processes in the oral cavity may be a risk factor for vascular diseases [1, 4]. There is strong evidence for a statistically significant dependence between the poor state of health in the oral cavity and the incidence of some systemic diseases, especially endocarditis, coronary atherosclerosis, carotid atherosclerosis, abdominal aortic atherosclerosis, myocardial infarction, aortic aneurysms, rheumatoid arthritis, chronic rhinosinusitis and even preeclampsia [1, 4–16]. Currently, the systemic effects of marginal periodontitis are considered to be much more important than the effects of apical periodontitis [15]. Furthermore, it appears that periodontitis is a risk factor for acute myocardial infarction [17, 18]. Studies of different populations have indicated that atherosclerosis may be related to chronic oral infections due to a metastatic spread of infection after bacteremia, when endothelial dysfunction contributes to atherothrombosis [4, 16]. It is plausible that periodontitis may have an impact on cardiovascular diseases via translocated circulating microbiota, which may directly or indirectly induce systemic inflammation. It contributes to development of atherothrombogenesis. There is evidence that oral bacterial species can enter the circulation during daily life activities (i.e., toothbrushing) and oral interventions (i.e., scaling), especially in gingivitis and periodontitis patients [7]. The relationship between atherosclerosis, aneurysms and periodontitis may be due to (a) the direct influence of subgingival plaque bacteria on the formation of atherosclerotic plaques, (b) the indirect influence of the inflammatory process on the organism, (c) the presence of genetic predispositions to periodontal diseases and atherosclerosis and (d) the presence of risk factors for both disease entities [4, 6, 8, 17–24] (Fig 1).

There is also evidence of higher levels of C-reactive protein (CRP) in periodontitis patients than in healthy subjects and a decrease in CRP levels as an effect of periodontal therapy [25]. There are higher levels of fibrinogen and platelet activation markers in periodontitis patients than in healthy controls [25].

Antibodies from periodontal pathogens can cross-react with antigens in cardiovascular tissues and activate cytokine production and monocyte and endothelial cell activation [26]. Patients with periodontitis have elevated levels of total cholesterol, low-density lipoproteins (LDL), triglycerides, oxidized LDL and phospholipase A2 [26].

Endothelial damage to vessel walls by mechanical, metabolic or immunological factors may initiate pathological reconstruction. In cases of aneurysm formation, there are two possible mechanisms of vessel wall damage: direct damage to the aorta and impairment of blood supply due to septic microtraps to the vasa-vasorum. There is also an autoimmunological theory of aneurysm formation. Molecular mimicry may explain the existence of common epitopes for bacterial proteins and aortic matrix proteins. Thus, immunological reactions may lead to aneurysm formation.

The subgingival bacterial burden has an impact on salivary lipopolysaccharide levels, which contribute to endotoxemia and the systemic destruction of connective tissue [9]. One of the bacteria present on the oral plaque biofilm *P*. *gingivalis* is of particular interest with regard to vascular diseases and systemic implications [10, 13, 24]. Clinical investigations have demonstrated that periodontal pathogens accelerate the progression of aortic aneurysms, particularly the impact of *P*. *gingivalis*, which affects tissues through Toll-like receptors (TLRs) and matrix metalloproteinases (MMPs) [13]. Various studies have demonstrated the presence of *P*. *gingivalis* in atherothrombotic tissues and multiple bacteria, such as *Propionibacterium acnes*, *Propionibacterium granulosum*, *Actinomyces viscosus*, *Actinomyces naeslundii*, and *Eggerthella lenta*, in aortic aneurysms [23, 24, 27–30]. Common genetic factors associated with periodontitis, cardiovascular diseases, type 2 diabetes, and Alzheimer’s disease were identified by Aarabi et al. [31]. Offenbacher also described common risk factors for periodontitis and atherosclerosis and introduced the term “PAS syndrome” (periodontitis-atherosclerosis syndrome). Patients who qualified for the PAS group were described as 3:60, meaning that for 60% of examined teeth surfaces, the CAL value was at least 3 mm [21]. Some of the periodontopathogens in the structure of the atherosclerotic plaques were found to be *P*. *gingivalis*, *A*. *actinomycetemcomitans*, *P*. *intermedia*, *T*. *forsythia*, *T*. *denticola*, *Campylobacter rectus*, *Fusobacterium nucleatum* and *Eiknella corrodens*. The incidence of these bacteria ranged from 0 to 88%, as determined by molecular biological methods [24, 28, 30, 32–34]. On the other hand, the percentage of patients with a confirmed presence of microorganisms in aneurysmal walls ranged from 7% to 37% [7, 23, 24, 34]. Therefore, the presence of periodontopathogens in the vessel walls remains controversial.

The aim of the study was to evaluate the occurrence of 5 selected bacterial pathogens, *P*. *gingivalis*, *T*. *forsythia*, *A*. *actinomycetemcomitans*, *P*. *intermedia*, and *T*. *denticola*, in periodontal pockets and in vessel walls of aneurysms and atherosclerotic plaques in abdominal aorta and carotid arteries.

## Materials and methods

The prospective study included 39 adults treated for vascular diseases. Vessel sample collection was performed in the Vascular, Endovascular Surgery, Angiology and Phlebology Department of Poznan University of Medical Sciences before oral examination. The groups were the result of randomly planned surgeries in the department. Seventy-nine samples were collected, but 40 of these were excluded. Participation in the study was voluntary, and written informed consent was obtained from all the subjects. The study protocol was approved by the Bioethics Committee (number 1270/04). Patients underwent periodontal examination after surgery. Patients who were edentulous, suffered from diabetes and had undergone antibiotic therapy in the last six months were excluded from the research. Table 1 shows the characteristics of the study group.

**Table 1:** The characteristics of the study groups.

Research group	Subgroups	Number of patients	Sex		Age (years)	
		n	female	male	mean	range
Patients with periodontitis	A1-AAAn	11	3	8	66.6	51–79
	A2-AAAth	19	3	16	56.7	44–68
	A3-CA	9	4	5	67.2	54–75

• A1-AAAn–patients with abdominal aortic aneurysms and concomitant periodontal disease

• A2-AAAth–patients with abdominal aortic atherosclerosis and concomitant periodontal disease

• A3-CA–patients with carotid atherosclerosis and concomitant periodontitis

All of the patients in this clinical trial who had concomitant periodontal disease underwent an evaluation based on the indices of the state of the periodontium. The recorded parameters included API (Approximal Plaque Index), BOP (Bleeding on Probing), PPD (Probing Pocket Depth) (registered from six sites of each tooth), CAL (Clinical Attachment Level) (registered from 6 sites of each tooth), furcation involvement in three grade scale measured with a Nabers probe [35], and tooth mobility according to the Entin three grade scale [36]. We also interviewed patients about the reason for tooth loss. The test specimens consisted of subgingival plaques and atherosclerotic plaques or specimens of aneurysmal walls collected during vascular surgery for carotid atherosclerosis, abdominal aortic atherosclerosis and abdominal aortic aneurysms. The subgingival plaque samples were gathered with sterile paper points according to the method described by Conrads [37]. We placed sterile paper points in the three deepest periodontal pockets for 10 seconds to avoid contaminating them with blood. Subsequently, they were placed in sterile Eppendorf test tubes and frozen at -20°C. Atherosclerotic plaques were also placed with sterile forceps in sterile Eppendorf test tubes during surgeries. Subgingival plaque samples were taken two to three days after surgeries; therefore, there was no risk that vessel walls could be contaminated with periodontal bacteria during sample collection.

The alkaline lysis method was used to isolate DNA from the subgingival plaque samples. The sterile filter containing material taken from the periodontal pocket was soaked with 20 μl 0.2 M NaOH, followed by incubation at 75°C for 5 minutes. After incubation, the solution was neutralized with 180 μL of 0.04 M TRIS-HCl, pH 7.5, and 5 μl of the resultant solution was collected for PCR. Sections of vessel walls or atherosclerotic plaques were prepared for PCR by DNA isolation using proteinase K. Approximately 100–200 mg of tissue was homogenized in liquid nitrogen in sterile mortars with 680 μl of SE buffer (75 mM NaCl, 25 mM EDTA, pH 8.0), 20 μl of proteinase K (10 mg/ml) and 100 μl of 10% SDS. Samples were mixed thoroughly and incubated for 16 hours at 55°C. They were then extracted with a mixture of phenol:chloroform:isoamyl alcohol (50:49:1), and the DNA was precipitated with isopropanol. The samples were stored at -20°C. Reference bacterial strains that were used in the PCR were sourced from the American Type Culture Collection (ATCC). Table 2 describes the reference bacterial strains according to ATCC that were used for PCRs.

**Table 2:** Description of reference strains according to ATCC.

Species of bacteria	ATCC–catalog number	Place of isolation
***Porphyromonas gingivalis***	Genomic DNA	Gingival sulcus
	*Porphyromonas gingivalis*	
	ATCC 33277D	
***Aggregatibacter actinomycetemcomitans***	Genomic DNA	Subgingival plaque
	*Haemophilus actinomycetemcomitans*	
	ATCC 700685	
***Tanarella forsythia***	Genomic DNA	Periodontal pocket
	*Tanarella forsythia*	
	ATCC 43037	
***Treponema denticola***	*Treponema denticola*	Subgingival plaque
	ATCC 33520	
***Prevotella intermedia***	*Prevotella intermedia*	Subgingival plaque
	ATCC 15032	

Experienced biotechnologists performed the whole procedure related to reference strains. Based on the literature, we chose starters for 5 bacterial species, and the 16S rRNA gene was amplified; data were based on publications by Fouad et al. [38] and Cairo et al. [30].

The PCR conditions were developed in pilot studies. Data from previously published studies were used to select primers for the five bacterial species and to amplify the 16S rRNA gene (Table 3).

**Table 3:** Primer sequences. Primer sequences F–forward, R–reverse, PCR product size, annealing temperature (T_m_), number of PCR cycles, reference source [30, 38].

Pathogen	Product size	Primer sequence 5’- 3’		T_m_ (cycle number)	References
*Porphyromonas gingivalis*	405 bp	F	AGG CAG CTT GCC ATA CTG CG	60°C (35)	Fouad et al. [38]
		R	ACT GTT AGC AAC TAC CGA TGT		
*Tanarella forsythia*	746 bp	F	TAC AGG GGA ATA AAA TGA GAT ACG	60°C (35)	Fouad et al. [38]
		R	ACG TCA TCC CCA CCT TCC TC		
*Treponema denticola*	316 bp	F	TAA TAC CGA ATG TGC TCA TTT ACA T	55°C (35)	Fouad et al. [38]
		R	TCA AAG AAG CAT TCC CTC TTC TTC TTA		
*Prevotella intermedia*	259 bp	F	CGT GGA CCA AAG ATT CAT CGG TGG A	59°C (35)	Fouad et al. [38]
		R	CCG CTT TAC TCC CCA ACA AA		
*Aggregatibacter actinomycetem-comitans*	358 bp	F	AGC GGA CGT GAA AGA ACT TGC	68°C (35)	Cairo et al. [30]
		R	GCA ATA GGA ACC CCA TCT CTC AT		

Typically, the DNA was amplified from a volume of 20 μl. All of the components of the reaction mixture were mixed in one test tube. Next, 16 μl of the reaction mixture was transferred into PCR tubes with 4 μl of the DNA solution (50 ng/μl) from each patient. The reaction was always conducted on ice. The composition of the reaction mixture remained unchanged for all PCR analyses. Standard PCR was conducted in 20 μl with 200 ng DNA, 50 mM KCl, 10 mM Tris-HCl (pH 8.3), 1.5 mM MgCl_2_, 0.25 mM dNTPs, 7.5 pmol of each primer and 0.5 Taq polymerase unit.

The reaction was conducted in an MJ thermocycler. Different reaction conditions were applied for each of the five bacterial species: initial denaturation at 94°C for 5 min, denaturation at 94°C for 45 s, annealing for 45 s, and elongation at 72°C for 95 s. In the case of *P*. *gingivalis* and *T*. *forsythiensis*, annealing was conducted at 60°C. *A*. *actinomycetemcomitans* annealing was conducted at 68°C, *P*. *intermedia* annealing at 59°C and *Treponema denticola* annealing at 55°C. The PCR products were separated in a 1.5% agarose gel. After separation for 30 min at 100 V in TBE buffer, the DNA bacterial fragments were observed: for *P*. *gingivalis–* 405 base pairs, for *T*. *forsythiensis–* 746 base pairs, for *A*. *actinomycetemcomitans—*358 base pairs, for *P*. *intermedia*—259 base pairs, and for *T*. *denticola*—316 base pairs, respectively [39].

The results were reported as the mean ± SD. Mean values, standard deviations, coefficients of variation (%), and minimum and maximum values were calculated for different clinical indices to determine the measures of location and dispersion observation. The normality of the data was checked with the Shapiro–Wilk test. The data were analyzed using one-way ANOVA or Kruskal–Wallis test with post hoc tests (Tukey’s or Dunn’s, respectively). The data were also analyzed using Student’s t-test, Cochran-Cox’s test or Mann–Whitney’s test. In all tests, a p value <0.05 was considered to be significant. All analyses were carried out using STATISTICA v.13 (TIBCO Software Inc.).

## Results

### Results of the clinical periodontal investigation

**Fig 2:**
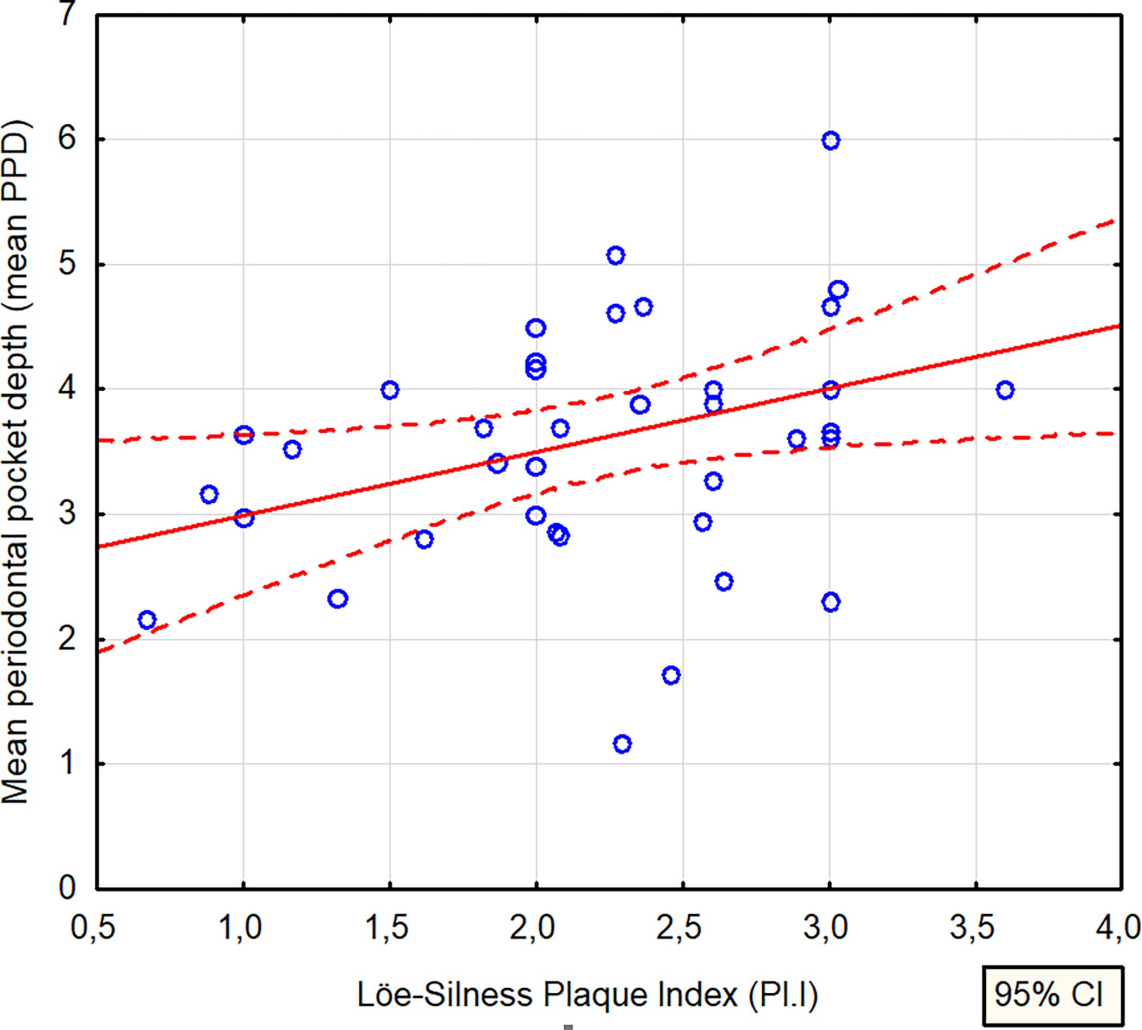
Chronic periodontitis advancement in the study subgroups, S-severe Periodontitis- Stage III and IV, Md or M- Moderate or Mild Periodontitis-Stage II.

Based on the indices and clinical evaluations, chronic periodontitis was diagnosed in all 39 patients. It was found that 69.2% suffered from generalized severe chronic periodontitis in stage III and IV [40]. Fig 2 shows the advancement of periodontal disease in three vascular disease groups.

The average values for parameters relating to periodontal status of the examined patients are presented in Table 4.

**Table 4:** Clinical characteristic of the study group. Data are shown as the mean ± SD.

Characteristics of Group A	The Study Group Size = 39
Age, years	61.9±9.2
Loe-Silness Pl.I (Plaque Index)	2.22±0.7
API	94%±11%
Loe-Silness GI (Gingivial Index)	1.68±0.6
BOP	90%±18%
Max PPD	5.9±1.9
Mean PPD	3.6±1.0
Max CAL	8.5±2.7
Mean CAL	5.7±2.4
Maximum tooth mobility	1.5±1,1
Maximum furcation involvement	0.76±1.0
Percantage of teeth surfaces with CAL ≥3 mm	82%±26%
Number of teeth	10.5±7.0

The periodontal parameters examined in the three groups due to vascular disease are presented in Table 5.

**Table 5:** The differences in periodontal status in subgroups AAAn, AAAth, and CA.

Parameter	AAAn (n = 11)			AAAth (n = 19)			CA (n = 9)			p
	X±SD	(min;max)	(Q_1,_Q_2_)	X±SD	(min;max)	(Q_1,_Q_2_)	X±SD	(min;max)	(Q_1,_Q_2_)	
Age	66±9.5	(51; 79)	(58; 75)	57±7	(44;68)	(51; 65)	67±7	(54;75)	(68;71)	P = 0.0023
PLI	2.1±0.8	(0.6; 3.0)	(1.3; 2.6)	2.33±0.7	(1.0; 3.6)	(2.0; 3.0)	2,1±0.6	(0.9; 3.0)	(68; 71)	P = 0.62
BOP	87%±23%	(40%; 100%)	(86%; 100%)	90%±19%	(23%; 100%)	(83%; 100%	96%±11%	(66%; 100%)	(100%; 100%)	P = 0.48
API	93%±14%	(55%; 100%)	(96%; 100%)	97%±7%	(71%; 100%)	(100%; 100%)	91%±13%	(71%,100%)	(77%; 100%)	P = 0.64
Mean PPD	3.1±0.7	(2.1; 10.0)	(4.0; 6.0)	3.6±1.1	(1.1; 6.0)	(2.9; 4.5)	4.1±0.9	(3.0; 6.0)	(3.4; 4.6)	P = 0.12
Mean CAL	4.1±1.3	(1.3; 5.3)	(3.0; 52)	6.5±2.8	(1.7; 11.0)	(4.3; 8.6)	6.2±2.1	(2.9; 10.0)	(5.9+7.1)	P = 0.037
Max PPD	5.1±1.8	(3.0; 10.0)	(4.0:6.0)	6.2±2.1)	(1.2; 6.0)	(2.9; 4.5)	6.4±1.8	(3.0; 9.0)	(6.0; 8.0)	P = 0.10
Max CAL	7.2±2.7	(3.0; 12.0)	(5.0; 8.0)	9.1±2.5	(3.0; 12.0)	(8.0; 11.0)	9.3±2.6	(6.0; 15.0)	(8.0; 10.0)	P = 0.14
Max tooth mobility	1.1±0.9	(0.0; 3.0)	(0.0; 2.0)	2.0±1.0	(0.0,3.0)	(1.0:3.0)	1.3±1.2	(0.0; 3.0)	(0.0; 2.0)	P = 0.07
Number of teeth	10.27±7.1	(1; 21)	(5.0; 18.0)	10.0±7.1	(1; 25)	(4.0; 14.0)	10.2±7.22	(1.21)	(5.0; 15.0)	P = 0.99

The mean patient Pl.I value was 2.1± 0.80, whereas the mean API was 93%±11%. There were no statistically significant differences between the mean Pl.I and API values in the individual subgroups, and the values of coefficients of variation indicated poor (Pl.I) or inappropriate (API) states of oral cavity hygiene (Table 5). The mean BOP value in Group A was 90%±19%, whereas in subgroups A1-AAAn, A2-AAAth, and A3-CA, the BOP values were 87% ± 2.34%, 90.3% ± 1.93%, and 96%± 1.1%, respectively. These values indicated that bleeding occurred in most of the sites while the sulcus was being probed. There were no statistically significant differences between the groups. The mean periodontal pocket depth (mean PPD) was 3.67 ± 1.11. There were no statistically significant differences between the subgroups with respect to the mean values of periodontal pocket depths (mean PPD). However, the patients with carotid atherosclerosis (A3-CA) tended to have slightly deeper periodontal pockets (x = 4.1) than the patients with abdominal aortic atherosclerosis (x = 3.6) or those with aortic aneurysms (x = 3.1) (p = 0.12).

**Fig 3:**
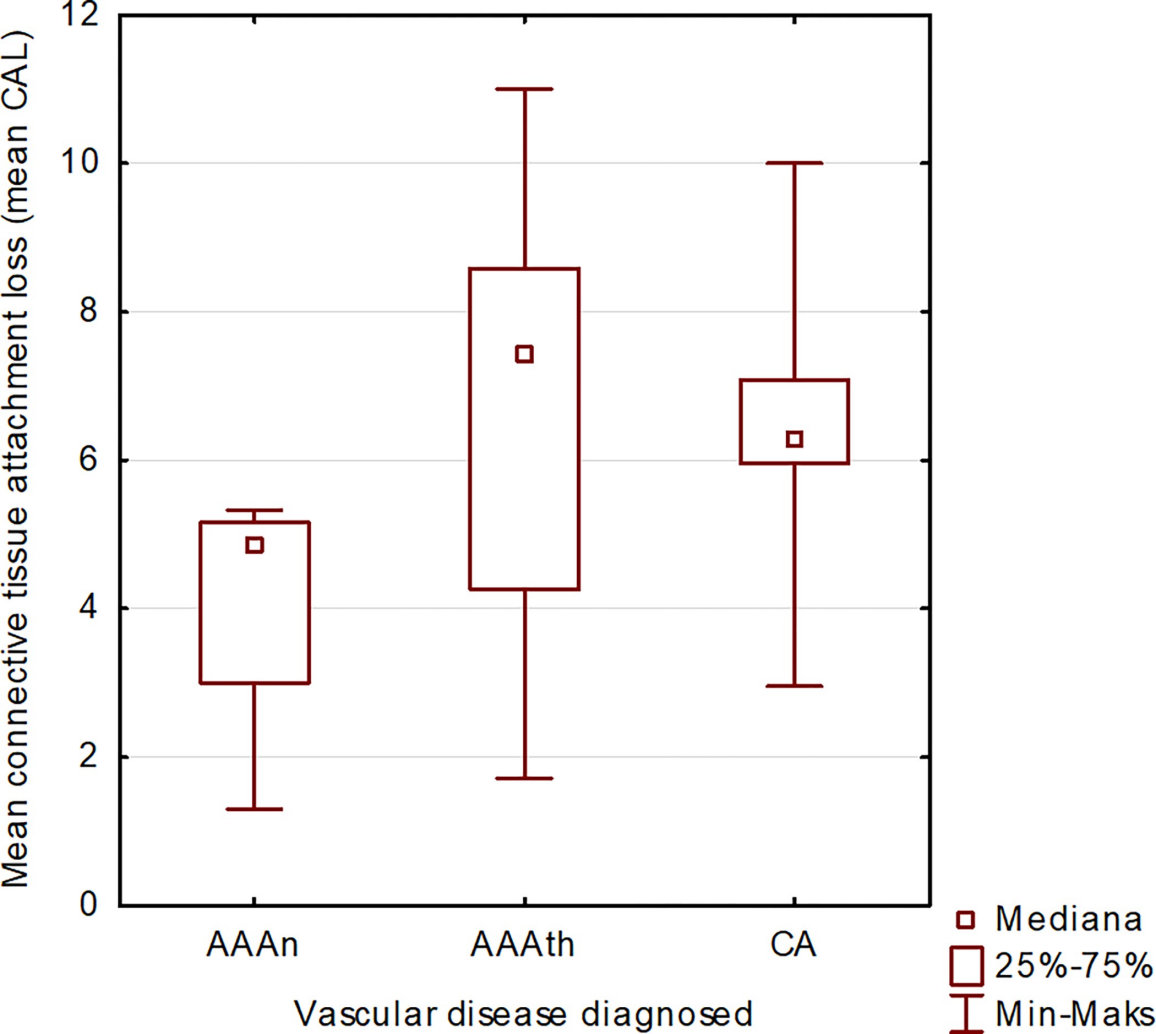
Mean PPD in correlation with Pl.I.

We confirmed correlation between the Pl.I and the mean PPD (Fig 3).

**Fig 4:**
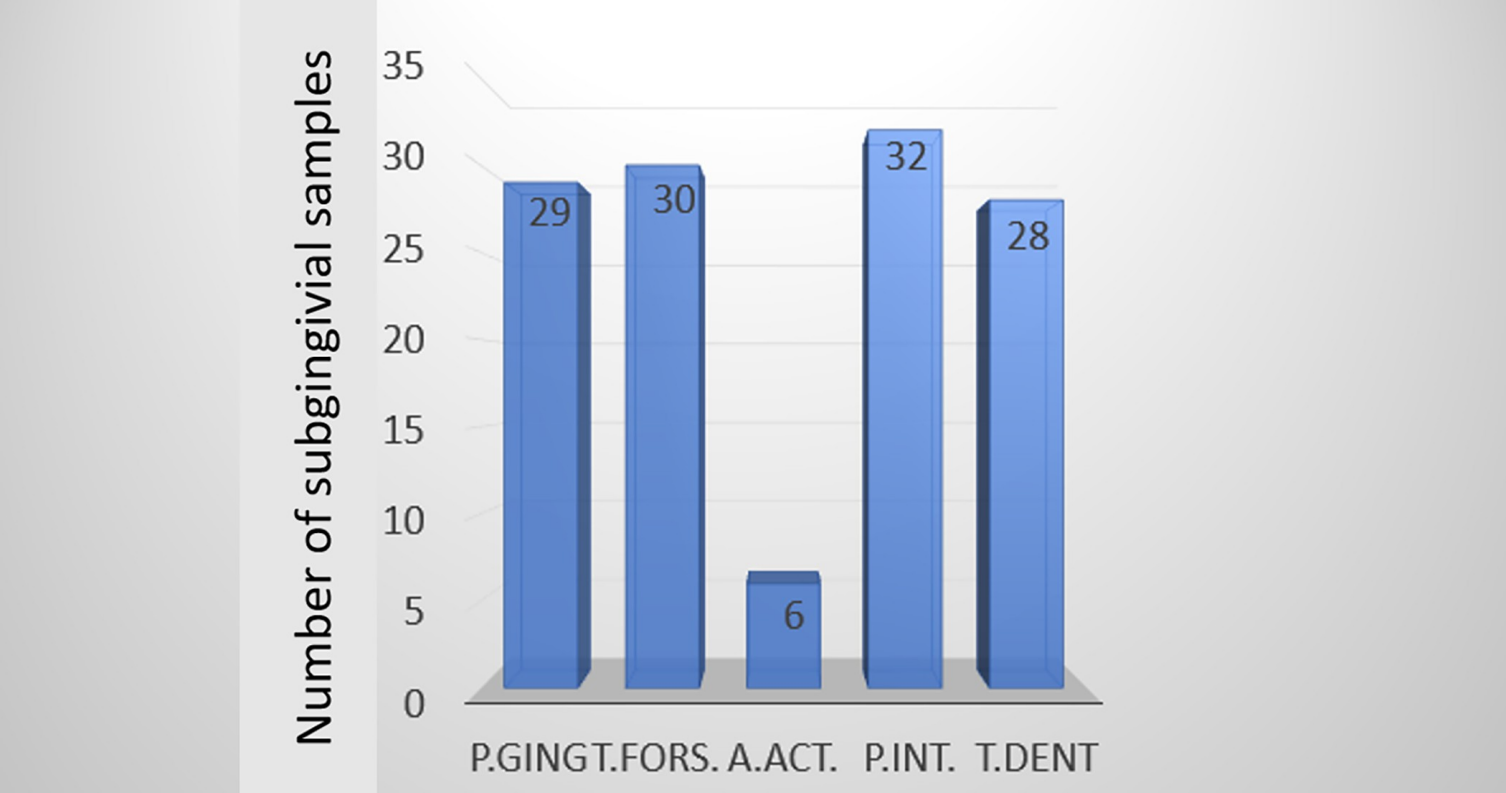
Mean CAL in Subgroups.

The mean value of the clinical attachment level was high (CAL): 5.81 ± 2.53. The statistical analysis revealed that the mean clinical attachment level values in patients from Groups A2-AAAth and A3-CA were significantly greater (p = 0.037) than the mean clinical attachment level values in patients from the A3-AAAn group (Fig 4).

The percentage of patients with 60% of teeth with CAL ≥ 3 mm was 84.6%, which suggests the prevalence of PAS syndrome (periodontitis-atherosclerosis syndrome) as described by Offenbacher et al. [21] (Table 6).

**Table 6:** The prevalence of periodontitis-atherosclerosis syndrome in the AAAn, AAAth, and CA groups.

Group		Patients with 60% or more teeth with CAL≥ 3 mm	Patients with fewer than 60% teeth with CAL≥ 3 mm
	n	n (%)	n (%)
	39	33 (84.6%)	6 (15.4%)
AAAn	11	9 (81.8%)	2 (8.2%)
AAAth	19	16 (84.2%)	3 (15.8%)
CA	9	8 (88.9%)	1 (11.1%)

### Results of laboratory PCR investigations

**Fig 5:**
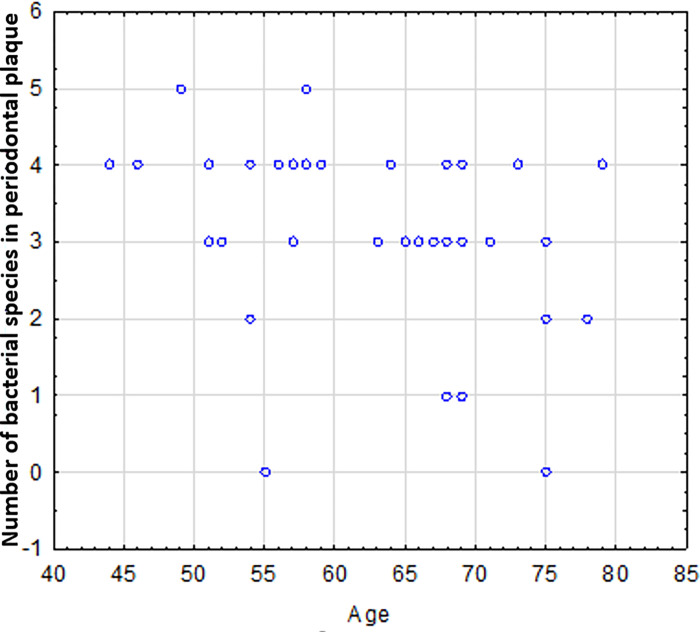
The prevalence of 5 bacterial species in subgingival plaque samples.

In total, 195 PCR analyses were undertaken to identify the 5 bacterial species in 39 subgingival plaque samples. The presence of at least one of these bacterial species was confirmed in 37 patients (94.9%). In two cases, none of the periodontopathogens under study were detected in the subgingival plaque. The most prevalent bacteria in subgingival plaque was *P*. *intermedia*, the least prevalent being *A*. *actinomycetemcomitans* (Fig 5).

**Fig 6:**
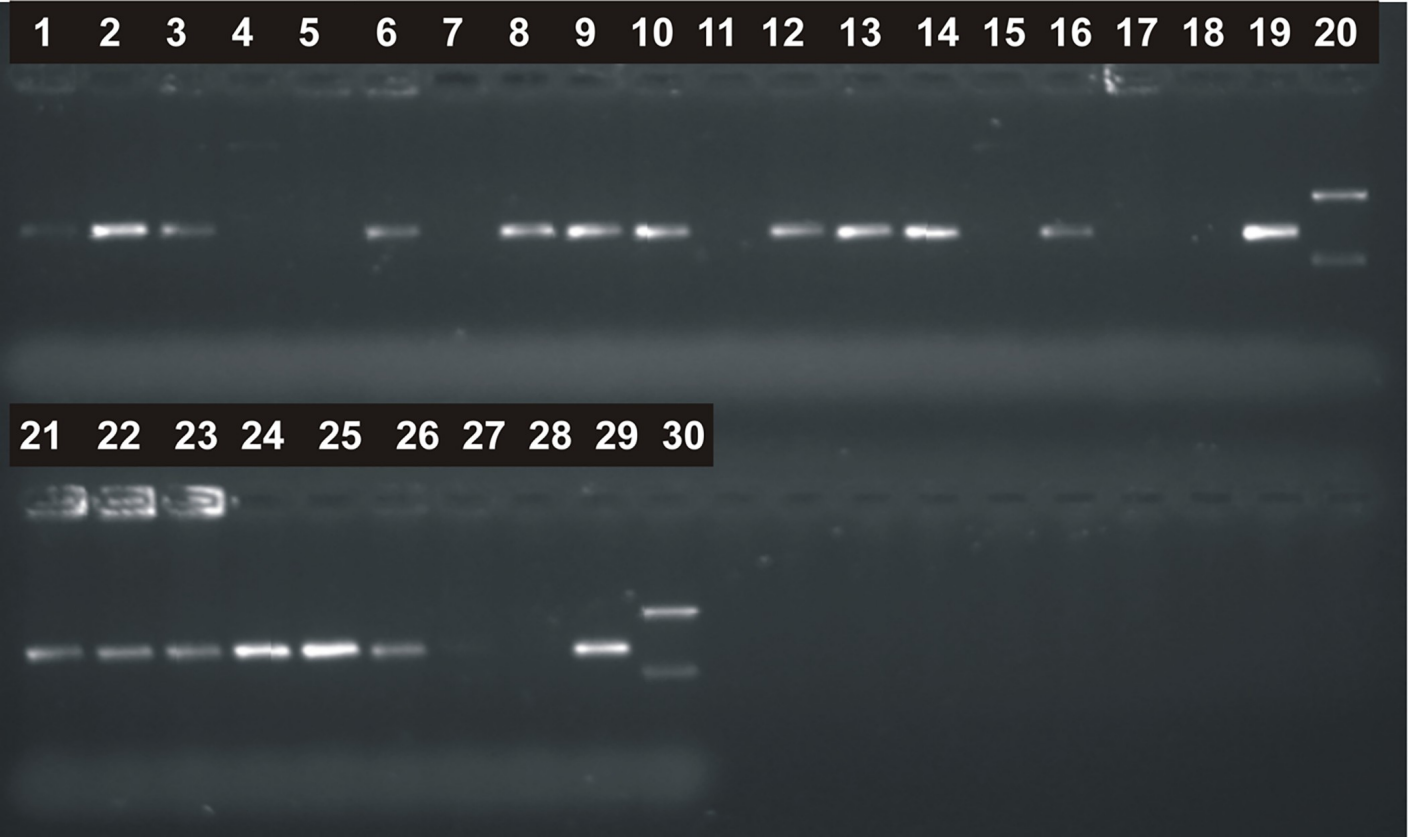
The prevalence of the 5 bacterial species with respect to the age of the patient.

We found that as patient age increased, fewer of the 5 bacterial species were detected in the plaque samples (Fig 6).

**Fig 7:**
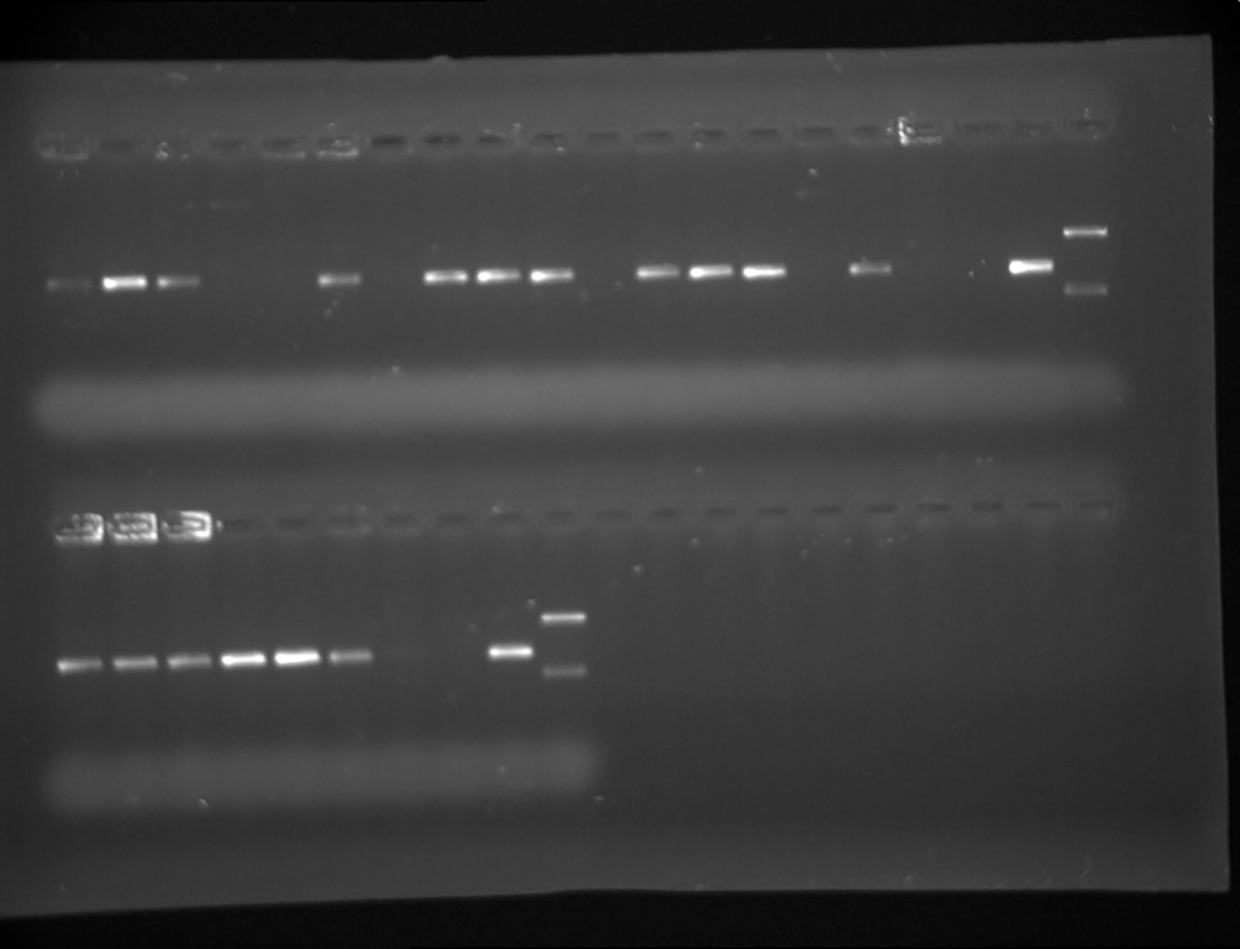
The separation of a PCR product for *P*. *gingivalis* for subgingival plaque samples.

The distribution of the PCR products in agarose gels of *P*. *gingivalis* collected from 24 subgingival plaque samples is presented in Fig 7.

PCR was conducted with *P*. *gingivalis* F and R primers, and the expected product size was 405 base pairs. Tracks 1–17 were subgingival plaque samples 40001–40017 (five digit numbers correspond to the patient identification). Tracks 21–30 were subgingival plaque samples 40018–40024. Tracks 18 and 28 were negative controls, with no DNA. Tracks 19 and 29 were positive control, PCR product (405 bp) from DNA *P*. *gingivalis* reference strain (ATCC 33277D). Tracks 20 and 30 were the size marker, 745 and 267 bp. PCR products were obtained for samples 40001, 40002, 40003, 40006, 40008, 40009, 40010, 40012, 40013, 40016, 40018, 40019, 40020, 40021, 40022, and 40023. However, there were no PCR products for samples 40004, 40005, 40007, 40011, 40017, and 40024.

There were simultaneous incidences of *P*. *gingivalis*, *T*. *f*o*rsythia* and *T*. *denticola* in 20 (51.3%) of the 39 patients. The three species form a red complex, which is thought to be most pathogenic to the periodontium. These were found in 14 patients with severe chronic periodontitis and 8 patients with moderate chronic periodontitis. The investigations showed a statistically significant dependence between the incidence of *P*. *gingivialis* and *T*. *forsythiensis* and the maximum periodontal pocket depths (p<0.05). *P*. *gingivialis* was the only periodontopathogen confirmed both in the atherosclerotic plaques and in the specimens of the vascular walls of patients with periodontitis. Neither of the remaining four bacterial periodontopathogens were detected in the vessel specimens. The presence of *P*. *gingivalis* was identified in a vascular wall specimen of only one patient from group AAAn, a 63-year-old smoker with a diagnosis of generalized severe chronic periodontitis. There were three species of periodontopathogens in the periodontal pockets of this patient: *P*. *gingivalis*, *P*. *intermedia* and *T*. *denticola*.

**Fig 8:**
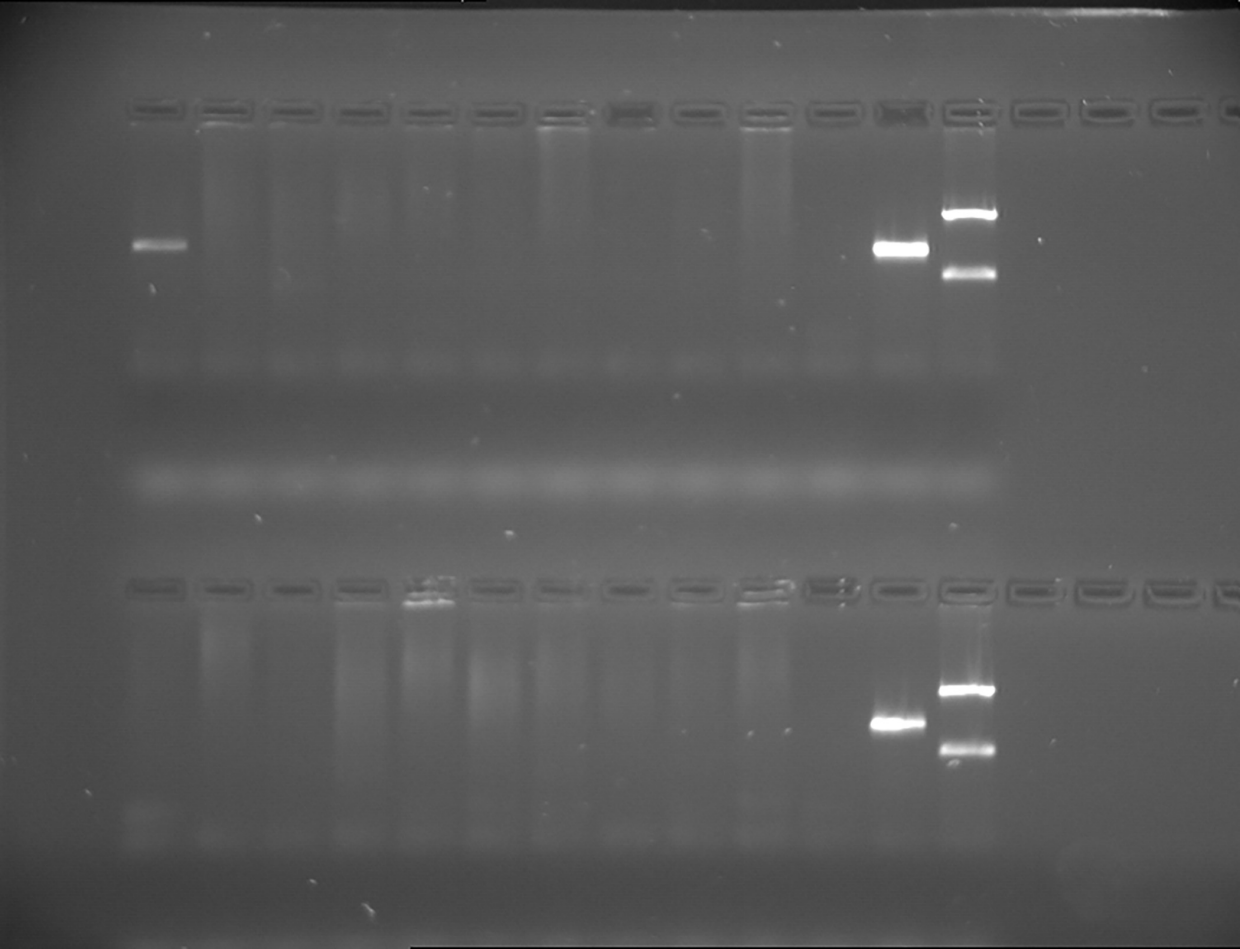
The distribution of a PCR product for *P*. *gingivalis* for atherosclerotic plaques.

Fig 8 shows a photograph of the distribution of vascular specimens.

A total of 20 atherosclerotic plaques were analyzed. PCR was conducted with *P*. *gingivalis* F and R primers, and the expected product size was 405 base pairs. Tracks 1–10 were atherosclerotic plaques 40001–40003, 40005, 40010, and 40013–40017. Tracks 14–23 were atherosclerotic plaques 40020, 40022–40025, 40031–40032, 40034–40035, and 40037. Tracks 11 and 24 were negative controls, with no DNA. Tracks 12 and 25 were positive control for PCR product (405 bp) for the *P*. *gingivalis* reference strain (ATCC 33277D), and tracks 13 and 26 were size markers, 745 and 267 base pairs. PCR product was obtained only for sample 40001.

Among the 39 patients investigated, *P*. *gingivalis* was the only periodontopathogen whose presence was confirmed in one specimen from the aneurysm walls. None of the other four bacteria were observed in vessel walls. None of the selected species of periodontopathogens were found in atherosclerotic plaques collected from patients with abdominal aortic atherosclerosis and carotid atherosclerosis.

## Discussion

Chronic periodontitis is considered to be the most frequently occurring pathological process in the oral cavity that adversely affects soft and hard tissues supporting the teeth. It is described as an inflammatory disease induced and maintained by a polymicrobial biofilm formed on teeth based on polymicrobial synergy and dysbiosis Hypotheses [41]. Homeostasis between the periodontopathogens in the dental biofilm and the host response is disrupted due to a burst of activity of the microorganisms or an imbalanced host response [42]. The role of periodontal pathogens is, however, still unclear, and the variability in the inflammatory response is perplexing [26, 43–45]. Epidemiological data show that nearly 50% of adults aged 30 years or more are affected by periodontitis [46]. The significance of bacterial species in complex biofilm-host interactions has been a subject of discussion in many articles [41].

The periodontal status of the examined group clearly indicated poor oral cavity condition in all 39 patients. Mean tooth number was just 10,59. These results are compatible with other authors’ results–Joshipura et al. [47] indicated that the number of lost teeth and poor oral status increase the prevalence of coronary diseases. Epidemiological studies show that there is a positive correlation between periodontal diseases and coronary heart disease [48]. Patients with severe periodontal disease demonstrated an increased risk of the first coronary event.

In our study, poor oral hygiene status and periodontium health were confirmed by PLI, API and SBI indices (mean API = 94,9%, mean BOP = 90%). An increase in the amount of dental plaque and gingival sulcus bleeding may promote vessel endothelium damage and eventual bacterial penetration to the bloodstream [20]. Chronic severe periodontitis stages III and IV were diagnosed in 71,7% of patients. Within the examined group, the mean PPD was 3,61, and the mean CAL was 5,7. Bad oral health status can be considered unsatisfactory and may influence the prevalence of cardiovascular diseases.

The presence of bacteria in the vessel walls and atheromatous plaques may lead to the confirmation of the possible virulence of periopathogens and their role in the aetiopathogenesis of atherosclerosis and aneurysm formation. The potential role of virulence factors originating from the oral cavity has been described for more than a hundred years [14, 15]. Not only bacteria but also products from subgingival bacteria, such as their metabolites, endotoxins and inflammatory mediators produced in periodontal tissues, have been strongly linked to systemic diseases [5]. Numerous studies have examined the migration of bacteria from periodontal pockets to atheromatous plaques in coronary vessels, carotid arteries and the walls of aneurysms. Intracellular survival of these organisms with dissemination to distant sites has been previously described (the Trojan Horse approach) [22, 43].

The presence of periodontal bacteria in the bloodstream or in situ in vascular lesions may be a risk factor associated with aneurysmal disease progression [20]. *P*. *gingivalis*, a black-pigmented, gram-negative, asaccharolytic bacterium, has also been identified in the gingival capillaries of patients with chronic periodontitis and was shown to have a profound effect on both the quantity and composition of oral microbiota [49]. This occurs even at a low abundance, when the bacterium behaves as a potential community activist together with a variety of virulence factors, ultimately leading to the development of a periodontal disease. *P*. *gingivalis* may invade epithelial cells of the gingival mucosa and endothelial cells [50, 51]. However, the presence of periopathogens in the vessel walls remains controversial.

Five species of pathogens for identification in vessel walls and subgingival plaques were chosen because 3 belong to the red complex, which, according to Socransky, is the most prevalent in periodontal pockets, and its presence is an indication of disease progression. *P*. *gingivialis* is also the most common periopathogen responsible for the pathogenesis of atherosclerosis and has the largest affinity for endothelial vessel invasion [6, 52].

Seventy-nine vessel samples were collected, but 40 of them were excluded because the patients were edentulous. There was also a limitation within the study, as it was not possible to proceed with radiological examination due to the patients’ physical form. Therefore, bone loss could not be evaluated precisely. The methodology of vessel wall preparation consisted of freezing the samples in liquid nitrogen and homogenizing the material to analyze the whole vessel sample.

In this study, the presence of periopathogens in periodontal pockets and vascular walls confirmed the possible ingress of periodontal bacteria into the bloodstream and their subsequent participation in the initiation and progression of vascular diseases. The presence of large populations of these bacteria in the subgingival plaque may be treated as a risk indicator of periodontal disease progression [44]. In addition, *P*. *gingivalis* has the greatest affinity toward the vascular endothelium and is most commonly observed in diseased vascular walls [8, 24, 28, 32]. *P*. *intermedia*, from the orange complex, is also regarded as a bacterium indicative of the progression of periodontal disease [49]. *A*. *actinomycetemcomitans*, from the green complex, is one of the indicator bacteria involved in the early stages of periodontitis. However, only a few authors have confirmed the simultaneous presence of *A*. *actinomycetemcomitans* in the periodontium and vascular walls [7, 8]. Martin et al. [53] reported the presence of the DNA of *A*. *actinomycetemcomitans* (the gene sequence encoding leukotoxin), originating from the oral cavity in an aortic aneurysm wall.

Most studies concerning the presence of bacteria in pathologically changed vessel walls focus on atherosclerotic plaque analyses [24, 27, 29, 32]. Only some investigations deal with the presence of periopathogens in aneurysmal walls. Positive results in reference to the presence of different periodontal bacteria in vessel walls vary between 0% and 84% for aortic aneurysms and 0% and 88.5% for atherosclerotic carotid arteries and abdominal aortas [27, 28, 34]. Kurihara showed that the most often occurring pathogens present in vessel walls were *P*. *gingivalis and T*. *denticola*, isolated in 85% and 63%, respectively, of patients with aortic aneurysms. Another study of Ishihara concerning carotid arteries showed the presence of *P*. *gingivalis* for 21.6% and *T*. *denticola* for 5.9% of examined samples [32].

It is difficult to interpret these findings, as they do not unequivocally confirm the relationship between the presence of bacteria in the periodontium and vascular walls. The presence of *P*. *gingivalis* in the aneurysm wall of only one patient in our study does not necessarily exclude the correlation between the incidence of vascular diseases and periodontal diseases. Differences in the occurrence of periopathogens may be due to population, geographical and ethnic differences, sampling methods of atherosclerotic plaques and aneurysm wall specimens and the use of a different laboratory methodology [29, 44]. The methods used in the isolation of DNA from collected samples and the types of primers used in the reactions are further factors that may influence the results. In this study, the choice of an appropriate reaction method was validated by the use of reference strains in each PCR and in the anticipated distribution of products for these strains. Furthermore, the identification of pathogens in subgingival plaque samples collected from periodontal pockets confirmed the choice of the methodology. The results revealed the presence of periopathogens in 94.6% of the patients when the same primers were used under identical reaction conditions.

Many authors have noted the complexity of the relationship between coronary vessel diseases and periodontitis [24]. This dependence may also be affected by the age of the population under study [24]. Other authors were not able to confirm the consistent presence of typical oral cavity pathogens in atherosclerotic plaques from the carotid artery or abdominal aorta [30]. Differences in the incidence of individual bacterial species populating the vascular walls and subgingival plaque indicate that there is no direct universal correlation. Zaremba et al. [54] reported that despite the presence of periodontal pathogens in atherosclerotic plaques, there was no dependence between their presence in the periodontium and in diseased vessels. The sample was only 15 patients. The authors explain this by humoral and cell immunological response that may influence the amount of bacteria getting to the vessel walls. Higher amounts of bacteria in vessels may be caused by patient immunodeficiency.

## Conclusions

Our results indicate that 94.9% of patients with atherosclerosis and abdominal aortic aneurysms presented with chronic severe or moderate generalized periodontitis in stages III and IV. The clinical attachment level and the percentage of examined areas affected by the disease as a consequence of poor oral hygiene were consistent with the criteria of PAS, which describes the coexistence of vascular and periodontal diseases. The clinical examinations showed that 84.6% of patients fulfilled the criterion “3:60”, which indicates CAL ≥3 mm in more than 60% of examined areas. Additionally, 94.6% of periodontal pockets revealed the occurrence of at least one periodontopathogen, with the most frequently detected periodontopathogens being *P*. *intermedia*, *T*. *forsythia and P*. *gingivalis*. Detection of *P*. *gingivalis* in aneurysmal walls and atheromatous plaques can be considered occasional but ensures the need for examination of a larger number of patients.

## Supporting information

S1 Data (PDF)

S1 Raw images (PDF)

## References

[1] Kassebaum NJ, Smith AGC, Bernabé E, Fleming TD, Reynolds AE, Vos T, et al. Global, regional, and national prevalence, incidence, and disability-adjusted life years for oral conditions for 195 countries, 1990–2015: a systematic analysis for the global burden of diseases, injuries, and risk factors. J Dent Res. 2017;96: 380–387. doi: 10.1177/0022034517693566 28792274 PMC5912207

[2] Górska R, Pietruska M, Dembowska E, Wysokińska-Miszczuk J, Włosowicz M, Konopka T. Prevalence of periodontal diseases in 35–44 year-olds in the large urban agglomerations. Dent Med Probl. 2012;49: 19–27.

[3] Tonetti MS, Jepsen S, Jin L, Otomo-Corgel J. Impact of the global burden of periodontal diseases on health, nutrition and wellbeing of mankind: a call for global action. J Clin Periodontol. 2017;44: 456–462. doi: 10.1111/jcpe.12732 28419559

[4] Tonetti MS, Van Dyke TE. Periodontitis and atherosclerotic cardiovascular disease: consensus report of the Joint EFP/AAP Workshop on Periodontitis and Systemic Diseases. J Periodontol. 2013;84: S24–S29. 10.1902/jop.2013.1340019 29537596

[5] Jin LJ, Lamster IB, Greenspan JS, Pitts NB, Scully C, Warnakulasuriya S. Global burden of oral diseases: emerging concepts, management and interplay with systemic health. Oral Dis. 2016;22: 609–619. doi: 10.1111/odi.12428 26704694

[6] Sanz M, Marco Del Castillo A, Jepsen S, Gonzalez-Juanatey JR, D’Aiuto F, Bouchard P, et al. Periodontitis and cardiovascular diseases: consensus report. J Clin Periodontol. 2020;47: 268–288. doi: 10.1111/jcpe.13189 32011025 PMC7027895

[7] Wada K, Kamisaki Y. Roles of oral bacteria in cardiovascular diseases—from molecular mechanisms to clinical cases: involvement of *Porphyromonas gingivalis* in the development of human aortic aneurysm. J Pharmacol Sci. 2010;113: 115–119. doi: 10.1254/jphs.09r22fm 20501967

[8] Figuero E, Lindahl C, Marín MJ, Renvert S, Herrera D, Ohlsson O, et al. Quantification of periodontal pathogens in vascular, blood, and subgingival samples from patients with peripheral arterial disease or abdominal aortic aneurysms. J Periodontol. 2014;85: 1182–1193. doi: 10.1902/jop.2014.130604 24502612

[9] Liljestrand JM, Paju S, Buhlin K, Persson GR, Sarna S, Nieminen MS, et al. Lipopolysaccharide, a possible molecular mediator between periodontitis and coronary artery disease. J Clin Periodontol. 2017;44: 784–792. doi: 10.1111/jcpe.12751 28556187

[10] Fiorillo L, Cervino G, Laino L, D’Amico C, Mauceri R, Tozum TF, et al. *Porphyromonas gingivalis*, periodontal and systemic implications: a systematic review. Dent J (Basel). 2019;7: 114. doi: 10.3390/dj7040114 31835888 PMC6960968

[11] Potempa J, Mydel P, Koziel J. The case for periodontitis in the pathogenesis of rheumatoid arthritis. Nat Rev Rheumatol. 2017;13: 606–620. doi: 10.1038/nrrheum.2017.132 28835673

[12] Byun SH, Min C, Park IS, Kim H, Kim SK, Park BJ, et al. Increased risk of chronic periodontitis in chronic rhinosinusitis patients: a longitudinal follow-up study using a national health-screening cohort. J Clin Med. 2020;9: 1170. doi: 10.3390/jcm9041170 32325855 PMC7231281

[13] Suzuki J, Aoyama N, Aoki M, Tada Y, Wakayama K, Akazawa H, et al. High incidence of periodontitis in Japanese patients with abdominal aortic aneurysm. Int Heart J. 2014;55: 268–270. doi: 10.1536/ihj.13-301 24806388

[14] Kaur S, White S, Bartold M. Periodontal disease as a risk factor for rheumatoid arthritis: a systematic review. JBI Libr Syst Rev. 2012;10: 1–12. doi: 10.11124/jbisrir-2012-288 27820156

[15] Slots J. Focal infection of periodontal origin. Periodontol 2000. 2019;79: 233–235. doi: 10.1111/prd.12258 30892771

[16] Genco R, Offenbacher S, Beck J. Periodontal disease and cardiovascular disease: epidemiology and possible mechanisms. J Am Dent Assoc. 2002;133: 14s–22s. doi: 10.14219/jada.archive.2002.0375 12085720

[17] Shi Q, Zhang B, Huo N, Cai C, Liu H, Xu J. Association between myocardial infarction and periodontitis: a meta-analysis of case-control studies. Front Physiol. 2016;7: 519. doi: 10.3389/fphys.2016.00519 27867362 PMC5095113

[18] Cueto A, Mesa F, Bravo M, Ocaña-Riola R. Periodontitis as risk factor for acute myocardial infarction. A case control study of Spanish adults. J Periodontal Res. 2005;40: 36–42. doi: 10.1111/j.1600-0765.2004.00766.x 15613077

[19] Persson GR, Persson RE. Cardiovascular disease and periodontitis: an update on the associations and risk. J Clin Periodontol. 2008;35: 362–379. doi: 10.1111/j.1600-051X.2008.01281.x 18724863

[20] Scannapieco FA, Genco RJ. Association of periodontal infections with atherosclerotic and pulmonary diseases. J Periodontal Res. 1999;34: 340–345. doi: 10.1111/j.1600-0765.1999.tb02263.x 10685358

[21] Offenbacher S, Madianos PN, Champagne CM, Southerland JH, Paquette DW, Williams RC, et al. Periodontitis-atherosclerosis syndrome: an expanded model of pathogenesis. J Periodontal Res. 1999;34: 346–352. doi: 10.1111/j.1600-0765.1999.tb02264.x 10685359

[22] Chukkapalli SS, Easwaran M, Rivera-Kweh MF, Velsko IM, Ambadapadi S, Dai J, et al. Sequential colonization of periodontal pathogens in induction of periodontal disease and atherosclerosis in LDLRnull mice. Pathog Dis. 2017;75: ftx003. doi: 10.1093/femspd/ftx003 28104616 PMC5353996

[23] Schenkein HA, Papapanou PN, Genco R, Sanz M. Mechanisms underlying the association between periodontitis and atherosclerotic disease. Periodontology 2000. 2020;83: 90–106. doi: 10.1111/prd.12304 32385879

[24] Armingohar Z, Jørgensen JJ, Kristoffersen AK, Abesha-Belay E, Olsen I. Bacteria and bacterial DNA in atherosclerotic plaque and aneurysmal wall biopsies from patients with and without periodontitis. J Oral Microbiol. 2014;6: 23408. doi: 10.3402/jom.v6.23408 25006361 PMC4024159

[25] Demmer RT, Trinquart L, Zuk A, Fu BC, Blomkvist J, Michalowicz BS, et al. The influence of anti-infective periodontal treatment on C-reactive protein: a systematic review and meta-analysis of randomized controlled trials. PLoS One. 2013;8: e77441. doi: 10.1371/journal.pone.0077441 24155956 PMC3796504

[26] Schenkein HA, Loos BG. Inflammatory mechanisms linking periodontal diseases to cardiovascular diseases. J Clin Periodontol. 2013;40 Suppl 14: S51–S69. 23627334 10.1111/jcpe.12060 PMC4554326

[27] Kurihara N, Inoue Y, Iwai T, Umeda M, Huang Y, Ishikawa I. Detection and localization of periodontopathic bacteria in abdominal aortic aneurysms. Eur J Vasc Endovasc Surg. 2004;28: 553–558. doi: 10.1016/j.ejvs.2004.08.010 15465379

[28] da Silva RM, Lingaas PS, Geiran O, Tronstad L, Olsen I. Multiple bacteria in aortic aneurysms. J Vasc Surg. 2003;38: 1384–1389. doi: 10.1016/s0741-5214(03)00926-1 14681645

[29] Fiehn NE, Larsen T, Christiansen N, Holmstrup P, Schroeder TV. Identification of periodontal pathogens in atherosclerotic vessels. J Periodontol. 2005;76: 731–736. doi: 10.1902/jop.2005.76.5.731 15898933

[30] Cairo F, Gaeta C, Dorigo W, Oggioni MR, Pratesi C, Pini Prato GP, et al. Periodontal pathogens in atheromatous plaques. A controlled clinical and laboratory trial. J Periodontal Res. 2004;39: 442–446. doi: 10.1111/j.1600-0765.2004.00761.x 15491349

[31] Aarabi G, Zeller T, Seedorf H, Reissmann DR, Heydecke G, Schaefer AS, et al. Genetic susceptibility contributing to periodontal and cardiovascular disease. J Dent Res. 2017;96: 610–617. doi: 10.1177/0022034517699786 28530468

[32] Ishihara K, Nabuchi A, Ito R, Miyachi K, Kuramitsu HK, Okuda K. Correlation between detection rates of periodontopathic bacterial DNA in coronary stenotic artery plaque [corrected] and in dental plaque samples. J Clin Microbiol. 2004;42: 1313–1315. doi: 10.1128/JCM.42.3.1313-1315.2004 15004106 PMC356820

[33] Okuda K, Ishihara K, Nakagawa T, Hirayama A, Inayama Y, Okuda K. Detection of *Treponema denticola* in atherosclerotic lesions. J Clin Microbiol. 2001;39: 1114–1117. doi: 10.1128/JCM.39.3.1114-1117.2001 11230436 PMC87882

[34] Ding F, Lyu Y, Han X, Zhang H, Liu D, Hei W, et al. Detection of periodontal pathogens in the patients with aortic aneurysm. Chin Med J (Engl). 2014;127: 4114–4118. 25430459

[35] Tarnow D, Fletcher P. Classification of the vertical component of furcation involvement. J Periodontol. 1984;55: 283–284. doi: 10.1902/jop.1984.55.5.283 6588186

[36] Wolf HF, Rateitschak EM, Rateitschak KH. Periodontology. Lublin: Czelej; 2006.

[37] Conrads G. DNA probes and primers in dental practice. Clin Infect Dis. 2002;35: S72–S77. doi: 10.1086/341924 12173112

[38] Fouad AF, Barry J, Caimano M, Clawson M, Zhu Q, Carver R, et al. PCR-based identification of bacteria associated with endodontic infections. J Clin Microbiol. 2002;40: 3223–3231. doi: 10.1128/JCM.40.9.3223-3231.2002 12202557 PMC130810

[39] Słomski R. Przykłady analiz DNA [Examples of DNA analyses].1st ed. Poznan University of Agriculture Press; 2001

[40] Papapanou PN, Sanz M, Buduneli N, Dietrich T, Feres M, Fine DH, et al. Periodontitis: consensus report of workgroup 2 of the 2017 world workshop on the classification of periodontal and peri-implant diseases and conditions. J Periodontol. 2018;89: S173–S182. doi: 10.1002/JPER.17-0721 29926951

[41] Könönen E, Gursoy M, Gursoy UK. Periodontitis: a multifaceted disease of tooth-supporting tissues. J Clin Med. 2019;8: 1135. doi: 10.3390/jcm8081135 31370168 PMC6723779

[42] Oscarsson J, Johansson A. Comment from the editor to the special issue: “periodontitis: from dysbiotic microbial immune response to systemic inflammation”. J Clin Med. 2019;8: 1706. 10.3390/jcm8101706 PMC6832643 31623278

[43] Dahlen G, Basic A, Bylund J. Importance of virulence factors for the persistence of oral bacteria in the inflamed gingival crevice and in the pathogenesis of periodontal disease. J Clin Med. 2019;8: 1339. doi: 10.3390/jcm8091339 31470579 PMC6780532

[44] Socransky SS, Haffajee AD. Periodontal microbial ecology. Periodontol 2000. 2005;38: 135–187. doi: 10.1111/j.1600-0757.2005.00107.x 15853940

[45] Seinost G, Wimmer G, Skerget M, Thaller E, Brodmann M, Gasser R, et al. Periodontal treatment improves endothelial dysfunction in patients with severe periodontitis. Am Heart J. 2005;149: 1050–1054. doi: 10.1016/j.ahj.2004.09.059 15976787

[46] Thornton-Evans G, Eke P, Wei L, Palmer A, Moeti R, Hutchins S, et al. Periodontitis among adults aged ≥30 years—United States, 2009–2010. MMWR Suppl. 2013;62: 129–135. 24264502

[47] Joshipura KJ, Wand HC, Merchant AT, Rimm EB. Periodontal disease and biomarkers related to cardiovascular disease. J Dent Res. 2004;83: 151–155. doi: 10.1177/154405910408300213 14742654

[48] Dietrich T, Sharma P, Walter C, Weston P, Beck J. The epidemiological evidence behind the association between periodontitis and incident atherosclerotic cardiovascular disease. J Periodontol. 2013;84: S70–S84. doi: 10.1902/jop.2013.134008 23631585

[49] Salhi L, Rompen E, Sakalihasan N, Laleman I, Teughels W, Michel JB, et al. Can periodontitis influence the progression of abdominal aortic aneurysm? A systematic review. Angiology. 2019;70: 479–491. doi: 10.1177/0003319718821243 30596254

[50] Rajakaruna GA, Negi M, Uchida K, Sekine M, Furukawa A, Ito T, et al. Localization and density of Porphyromonas gingivalis and Tannerella forsythia in gingival and subgingival granulation tissues affected by chronic or aggressive periodontitis. Sci Rep. 2018;8: 9507. doi: 10.1038/s41598-018-27766-7 29934515 PMC6014976

[51] Mastragelopulos N, Haraszthy VI, Zambon JJ, Zafiropoulos GG. Detection of periodontal pathogenic microorganisms in atheromatous plaque. Preliminary results. Chirurg. 2002;73: 585–591. doi: 10.1007/s00104-001-0385-1 12149943

[52] Stelzel M, Conrads G, Pankuweit S, Maisch B, Vogt S, Moosdorf R, et al. Detection of *Porphyromonas gingivalis* DNA in aortic tissue by PCR. J Periodontol. 2002;73: 868–870. doi: 10.1902/jop.2002.73.8.868 12211495

[53] Martín MC, Andrés MT, Fierro JF, Méndez FJ. Endarteritis and mycotic aortic aneurysm caused by an oral strain of *Actinobacillus actinomycetemcomitans*. Eur J Clin Microbiol Infect Dis. 1998;17: 104–107. doi: 10.1007/BF01682165 9629975

[54] Zaremba M.; Górska R.; Suwalski P. Ocena występowania bakterii związanych z chorobą przyzębia w blaszce miażdżycowej naczyń wieńcowych / Assessment of the incidence of bacteria associated with periodontal disease in atherosclerotic coronary artery plaque. J of Stomatol. 2005;58(5): 293–301.

