# Supplementary material for: Periodontal status and the incidence of selected bacterial pathogens in periodontal pockets and vascular walls in patients with atherosclerosis and abdominal aortic aneurysms
Source: PLoS One. 2022 Aug 11;17(8):e0270177. doi: 10.1371/journal.pone.0270177 (PMC9371326; doi:10.1371/journal.pone.0270177)
Supplement: S1 Data — (PDF) [file pone.0270177.s001.pdf]

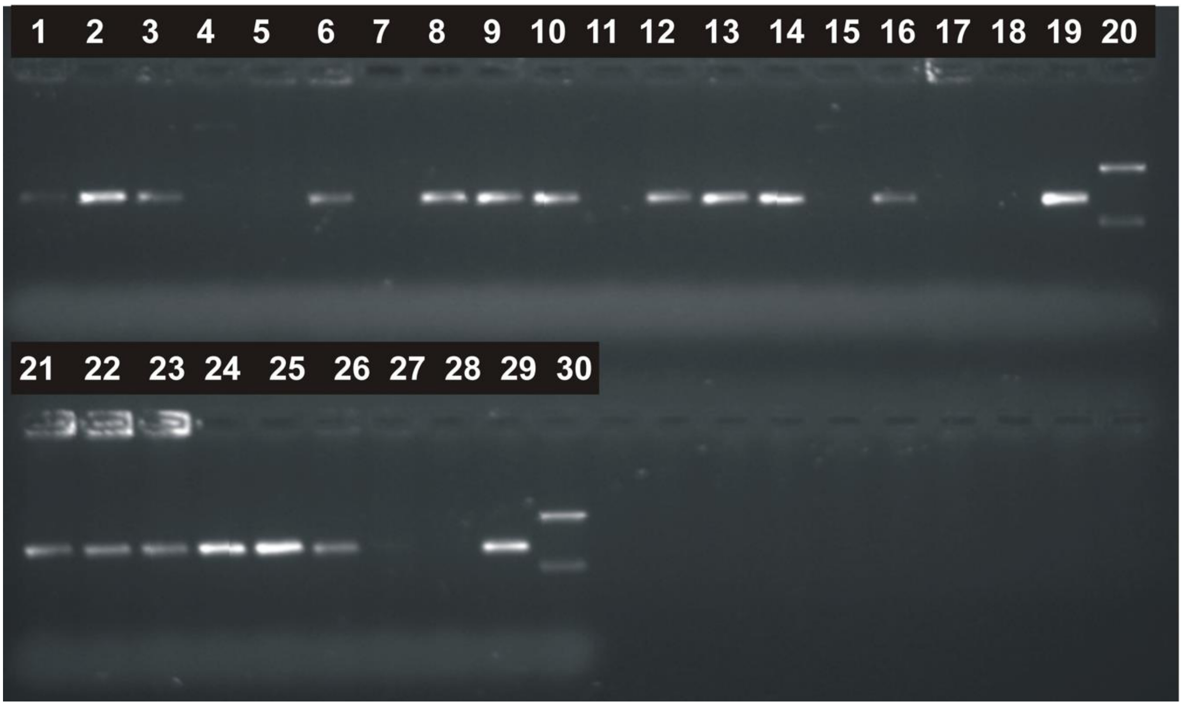

Fig.1. The separation of a PCR product in 1.5% agarose gel for *Porphyromonas gingivalis* for subgingival plaque samples. 24 subgingival plaque samples were analyzed. The PCR was conducted with *P. gingivalis* F and R primers. The expected product size was 405 base pairs. Lanes 1-17 were subgingival plaque samples 40001-40017; lanes 21-30 were subgingival plaque samples 40018-40024; lanes 18 and 28, negative control, no DNA; lanes 19 and 29, positive control, PCR product (405 bp) for *Porphyromonas gingivalis* reference strain DNA (ATCC 33277D); lanes 20 and 30, size marker, 745 and 267 bp. PCR products were obtained for samples: 40001, 40002, 40003, 40006, 40008, 40009, 40010, 40012, 40013, 40016, 40018, 40019, 40020, 40021, 40022, 40023. However, there were no PCR products for samples 40004, 40005, 40007, 40011, 40017, 40024.

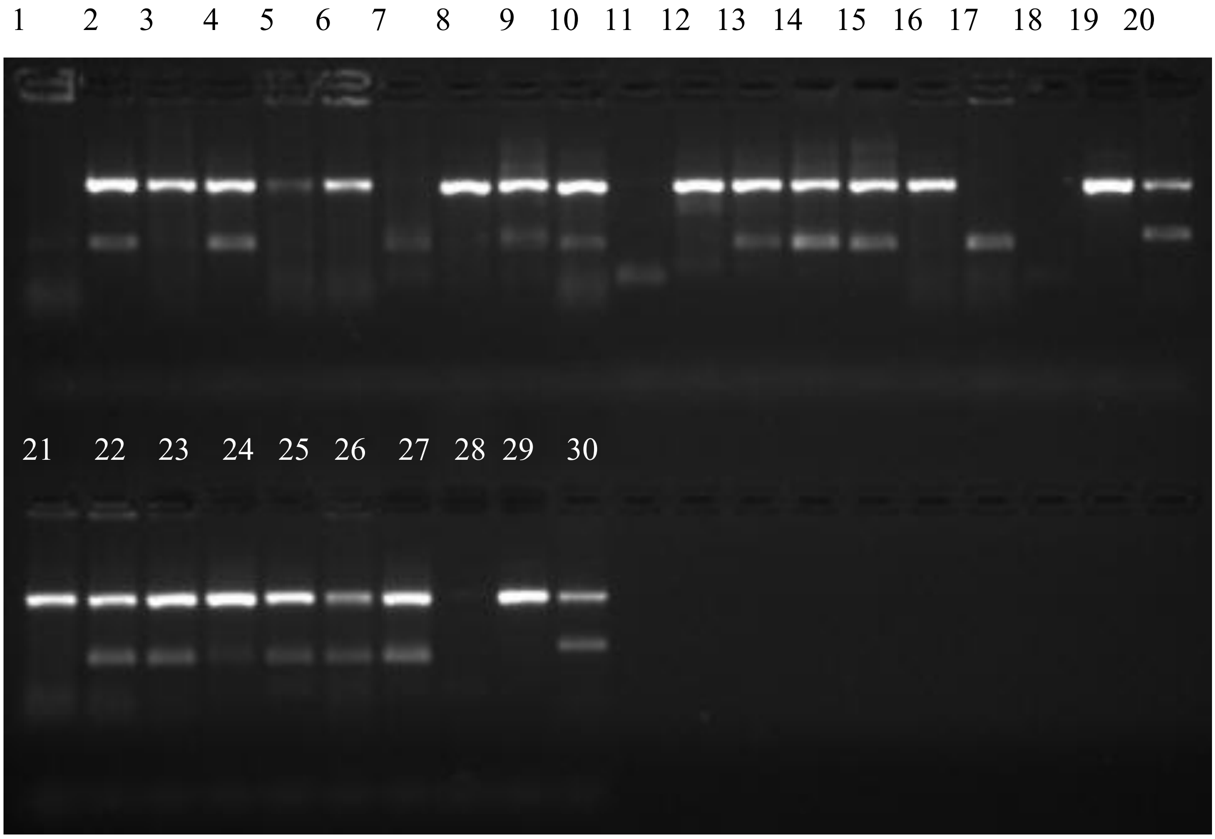

Fig.2. The separation of a PCR product in 1.5% agarose gel for *Tanarella forsythiensis* for subgingival plaque samples. 24 subgingival plaques samples were analyzed. The PCR was conducted with *T.fors*. F and R primers. The expected product size was 746 base pairs. Lanes 1-17 were subgingival plaque samples 40001-40017; lanes 21-30 were subgingival plaque samples 40018-40024; lanes 18 and 28, negative control, no DNA; lanes 19 and 29, positive control, PCR product for *Tanarella forsythiensis* reference strain DNA (ATCC 43037). PCR products were obtained for samples: 40002, 40003, 40004, 40005, 40006 ,40008, 40009, 40010, 40012, 40013, 40014, 40015, 40016, 40018, 40019, 40020, 40021, 40022, 40023, 40024. However, there were no PCR products for samples: 40001, 40007, 40011, 40017 .

1 2 3 4 5 6 7 8 9 10 11 12 13 14 15 16 17 18 19 20

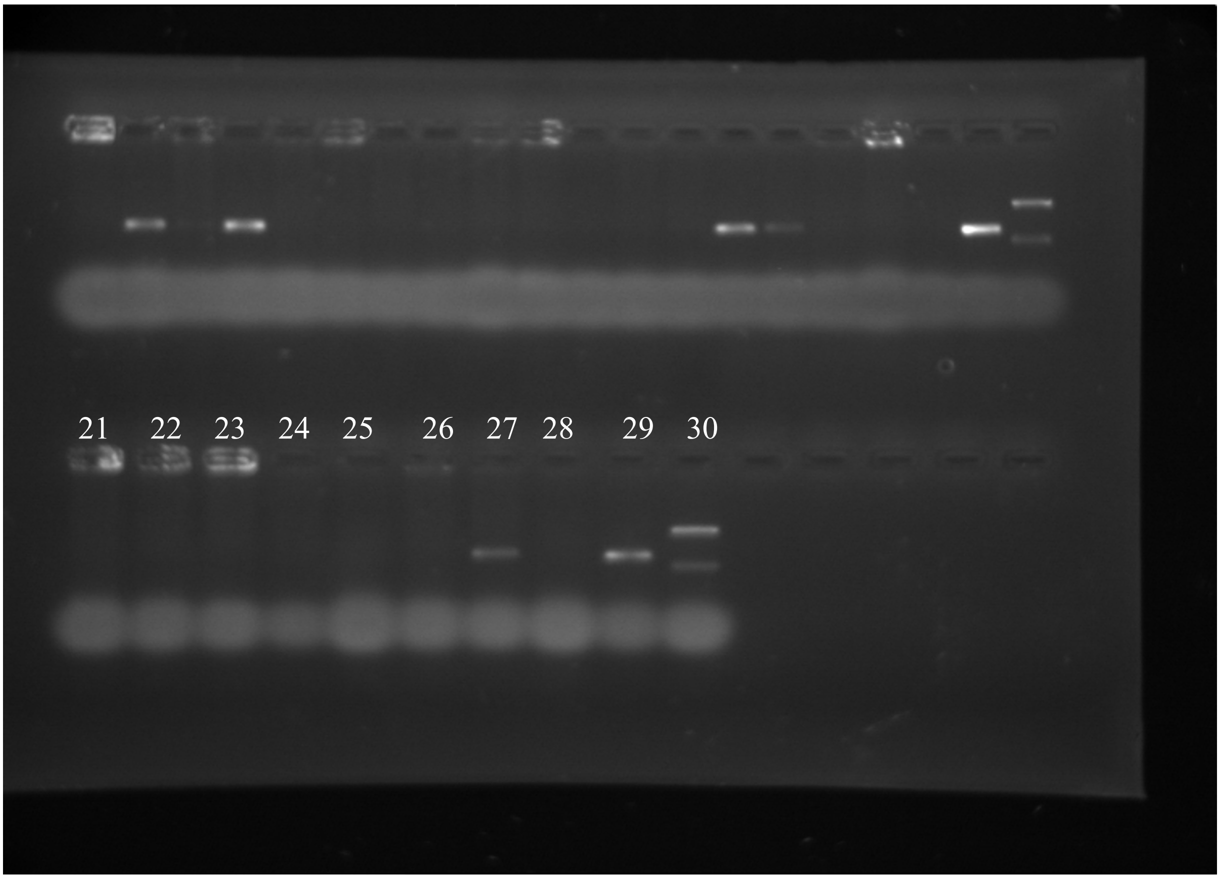

Fig.3. The separation of a PCR product in 1.5% agarose gel for *Actinomyces actinomycetemcomitans* for subgingival plaque samples. 24 subgingival plaque samples: 40001-40024 were analyzed. The PCR was conducted with *A.act.* F and R primers. The expected product size was 358 base pairs. Lanes 1-17 were subgingival plaque samples 40001-40017; lanes 21-30 were subgingival plaque samples 40018-40024; lanes 18 and 28, negative control, no DNA; lanes 19 and 29, positive control, PCR product for *Actinomyces actinomycetemcomitans* reference strain DNA (ATCC 700685). PCR products were obtained for samples: 40002, 40004, 40014, 40015, 40016, 40024; however, there were no PCR products for samples: 40001, 40003, 40005, 40006, 40007, 40008, 40009, 40010, 40011, 40012, 40013, 40014, 40017, 40018, 40019, 40020, 40021, 40022, 40023.

.

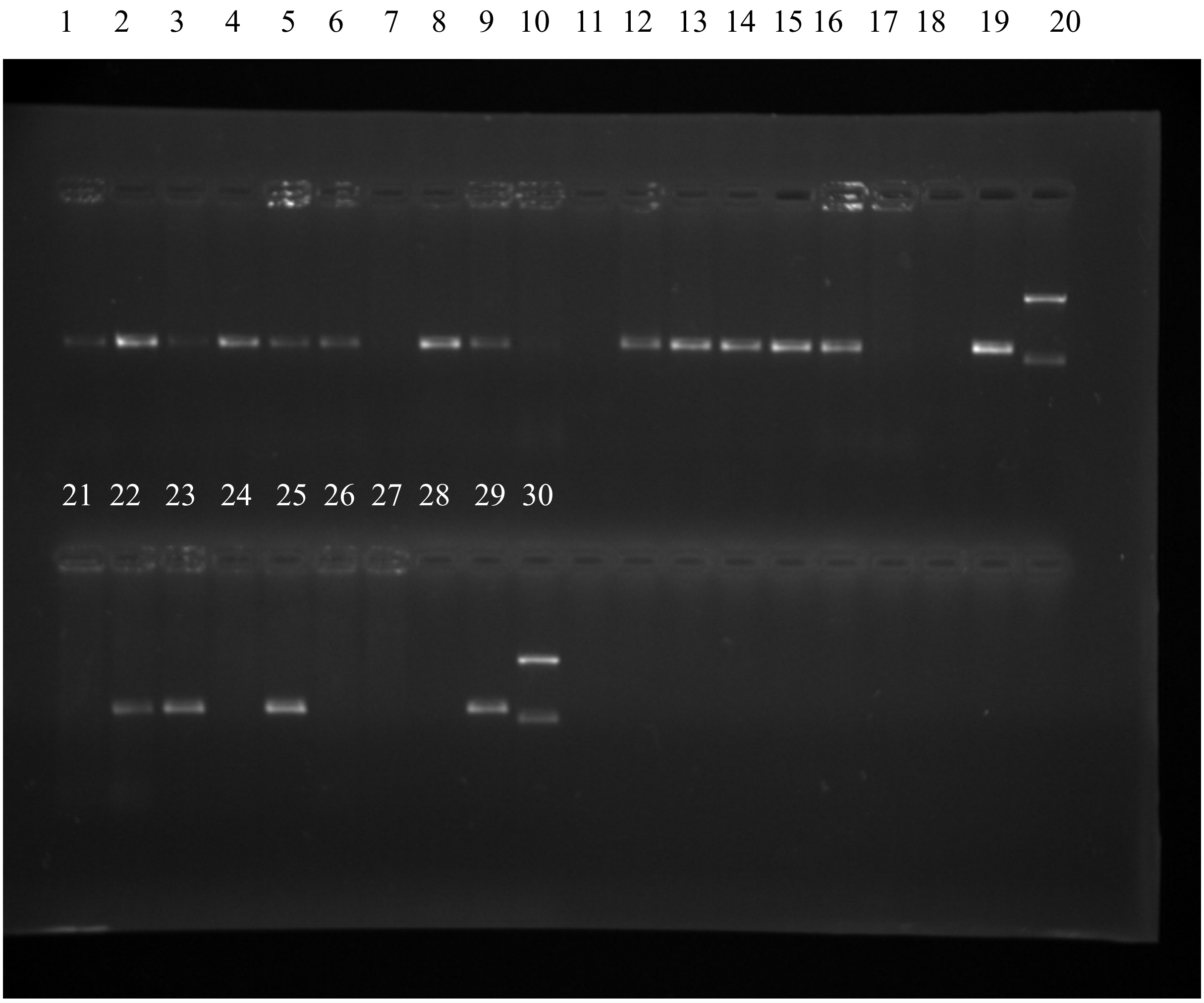

Fig.4. The separation of a PCR product in 1.5% agarose gel for *Treponema denticola* for subgingival plaque samples. 24 subgingival plaque samples were analyzed. The PCR was conducted with *T.dent.* F and R. primers. The expected product size was 316 base pairs. Lanes 1-17 were subgingival plaque samples 40001-40017; lanes 21-30 were subgingival plaque samples 40018-40024; lanes 18 and 28, negative control, no DNA; lanes 19 and 29, positive control, PCR product for *Treponema denticola* reference strain DNA (ATCC 33520). Products PCR were obtained for samples: 40001, 40002, 40003, 40004, 40005, 40006, 40008, 40009, 40012, 40013, 40014, 40015, 40016, 40019, 40020, 40022. However, there were no PCR products for samples: 40007, 40010, 40011, 40017, 40018, 40021, 40023, 40024.

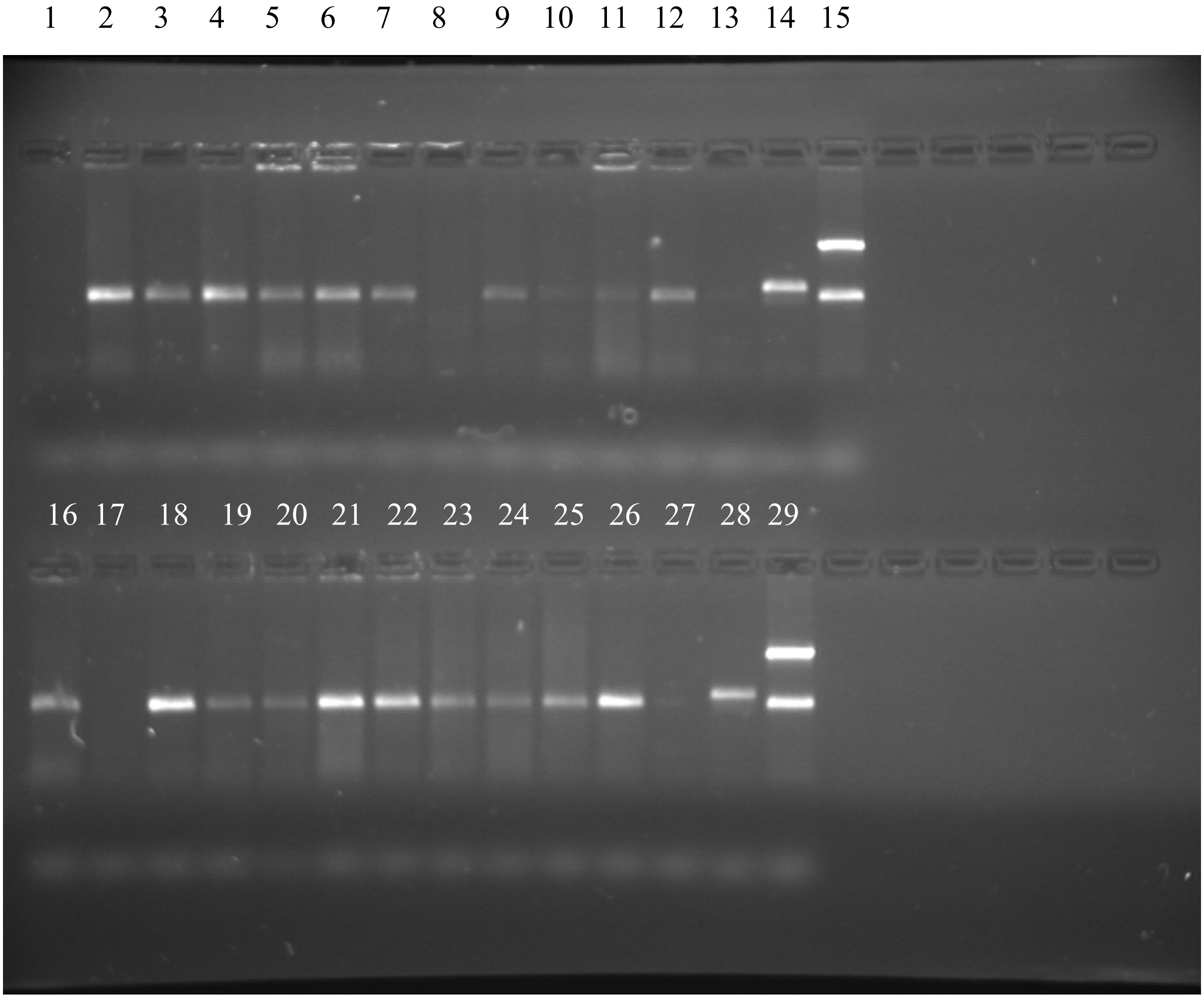

Fig.5. The separation of a PCR product in 1.5% agarose gel for *Prevotella intermedia* for subgingival plaque samples. 23 subgingival plaque samples were analyzed. The PCR was conducted with *P.int.* F and R primers. The expected product size was 259 base pairs. Lanes 1-12 were subgingival plaque samples 40078-40086, 40092-40093, 40096; lanes 16-26 were subgingival plaque samples 40098, 40109-40110, 40118, 40120, 40122, 40124-40128; lanes 13 and 27, negative control, no DNA; lanes 14 and 28, positive control, PCR product for *Prevotella intermedia* reference strain DNA (ATCC 15032). PCR products were obtained for samples: 40079, 40080, 40081, 40082, 40083, 40084, 40092, 40093, 40096, 40098, 40110, 40118, 40120, 40122, 40124, 40125, 40126, 40127, 40128; there were no PCR products for samples: 40078, 40085, 40086, 40109.

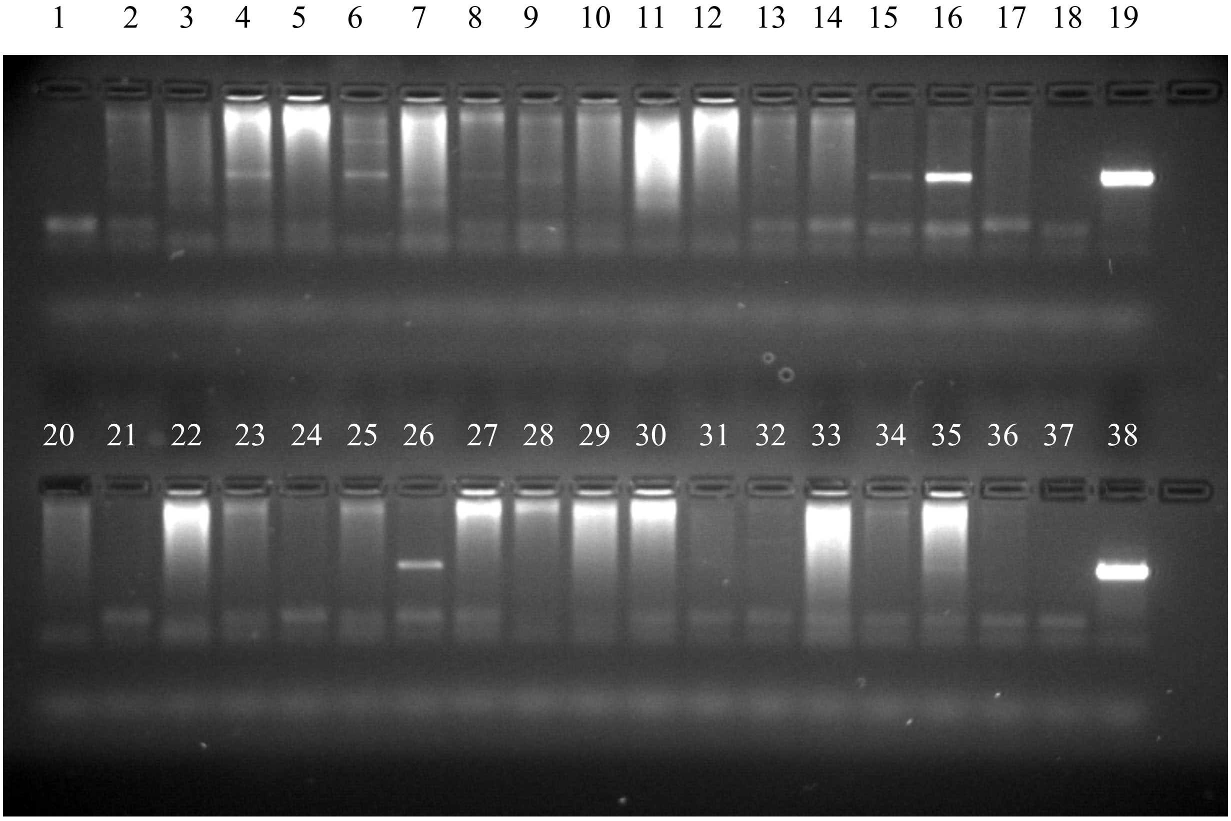

Fig.6. The separation of a PCR product in 1.5% agarose gel for *Porphyromonas gingivalis* for atherosclerotic plaques or aneurysm vessel walls. 34 atherosclerotic plaques were analyzed. The PCR was conducted with *P. ging* F and R. primers. The expected product size was 405 base pairs. Lanes 1-17 were atherosclerotic plaques 40082-40083, 40088-40096, 40097-400101; lanes 20-36 were atherosclerotic plaques 400102-40103, 40105-40108, 40113-40121, 40123-40125; lanes 18 and 37, negative control, no DNA; lanes 19 and 38, positive control, PCR product for *Porphyromonas gingivalis* reference strain DNA (ATCC 33277D). PCR products were obtained for samples:40089,40091,40099,40100,40113.

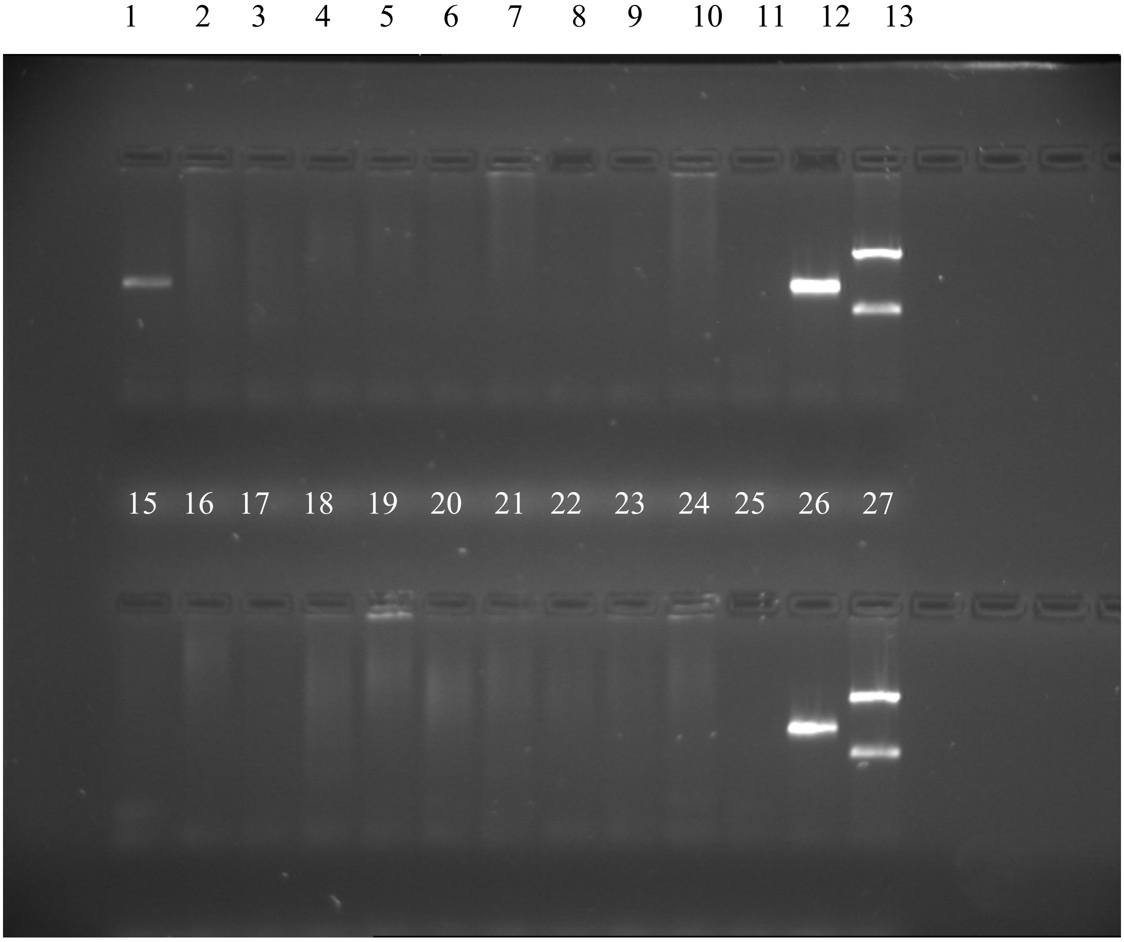

Fig.7. The separation of a PCR product in 1.5% agarose gel for *Porphyromonas gingivalis* for atherosclerotic plaques or aneurysm vessel walls. A total of 20 atherosclerotic plaques were analyzed. The PCR was conducted with *P. gingivalis* F and R primers. The expected product size was 405 base pairs. Lanes 1-10 were atherosclerotic plaques 40001-40003, 40005, 40010, 40013-40017.; lanes 14-23 were atherosclerotic plaques 40020, 40022-40025, 40031-40032, 40034-40035, 40037; lanes 11 and 24, negative control, no DNA; lanes 12 and 25, positive control, PCR product (405 bp) for *Porphyromonas gingivalis* reference strain DNA (ATCC 33277D), lanes 13 and 26, size marker, 745 and 267 base pairs. PCR product was obtained only for sample 40001.

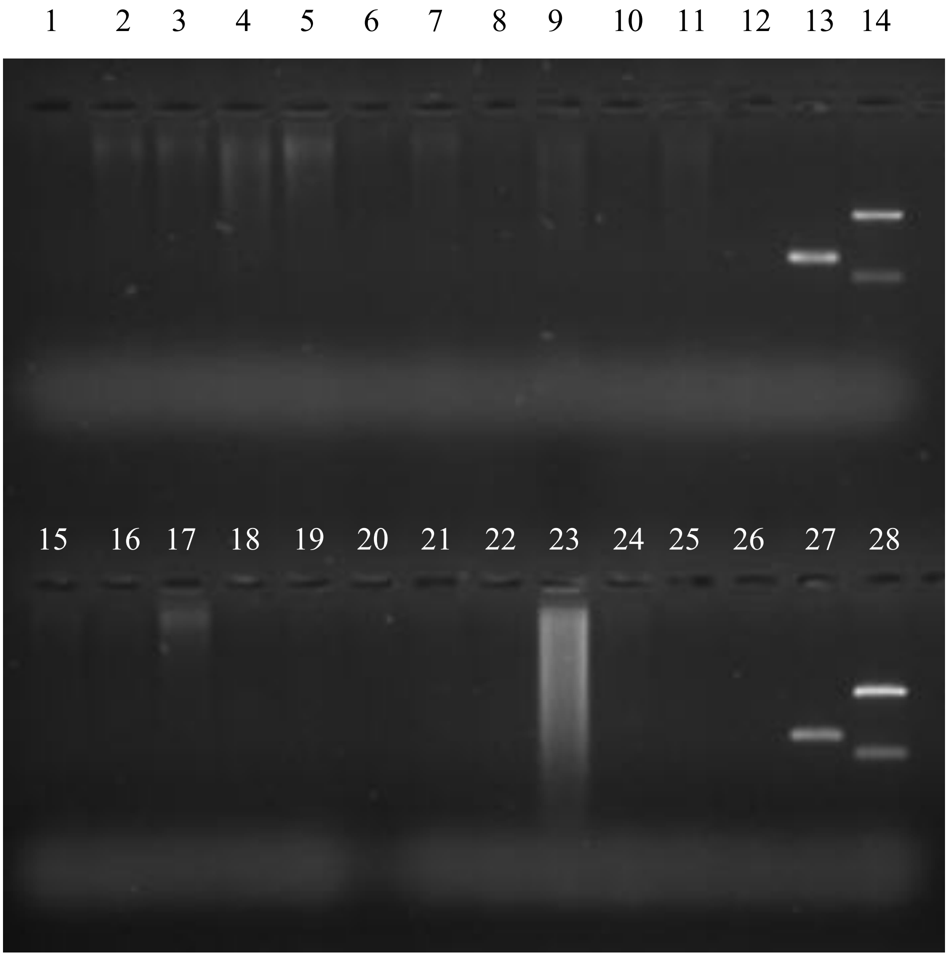

Fig.8. The separation of a PCR product in 1.5% agarose gel for *Actinomyces actinomycetemcomitans* for atherosclerotic plaques or aneurysm vessel walls.
22 atherosclerotic plaques were analyzed. The PCR was conducted with *A.act.* F and R. primers. The expected product size was 358 base pairs. Lanes 1-11 were atherosclerotic plaques 40082-40083, 40088-40096; lanes 15-25 were atherosclerotic plaques 40097-40103, 40105-40108; lanes 12 and 26, negative control, no DNA; lanes 13 and 27, positive control, PCR product for *Actinomyces actinomycetemcomitans* reference strain DNA (ATCC 700685). There was no PCR product for any of the samples.

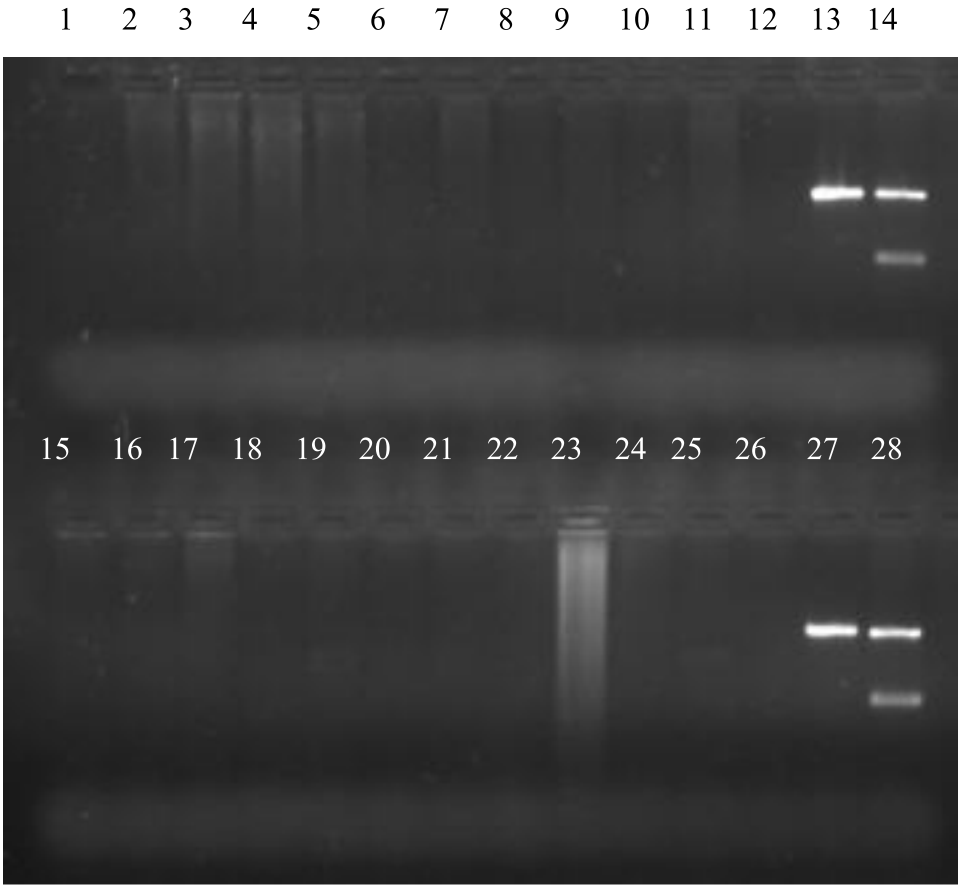

Fig.9. The separation of a PCR product in 1.5% agarose gel for *Tanarella forsythiensis* for atherosclerotic plaques or aneurysm vessel walls. 22 atherosclerotic plaques were analyzed. The PCR was conducted with *T.fors*. F and R primers. The expected product size was 746 base pairs. Lanes1-11 were atherosclerotic plaques 40082-40083, 40088-40096; lanes 15-25 were atherosclerotic plaques 40097-40103, 40105-40108; lanes 12 and 26, negative control, no DNA; tracks 13 and 27, positive control, PCR product for *Tanarella forsythiensis* reference strain DNA (ATCC 43037). There was no PCR product for any of the samples.

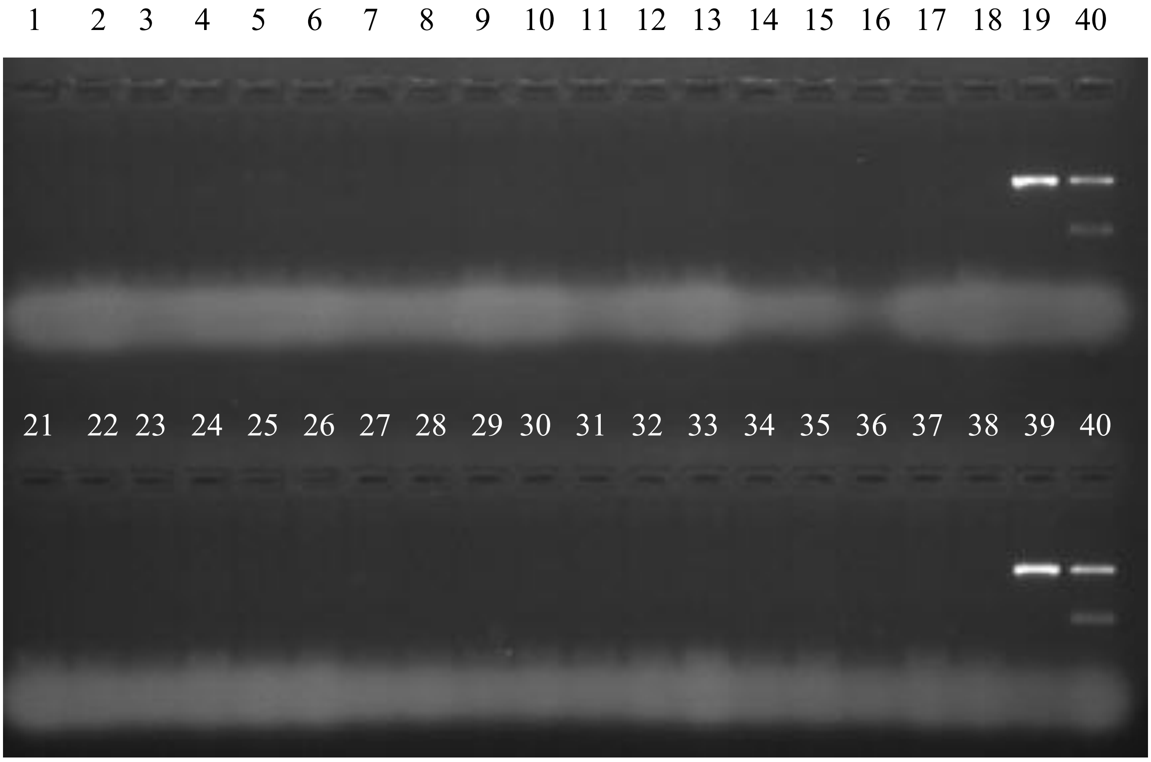

Fig.10. The separation of a PCR product in 1.5% agarose gel for *Tanarella forsythiensis* for atherosclerotic plaques or aneurysm vessel walls. 34 atherosclerotic plaques were analyzed. The PCR was conducted with *T.fors*. F and R primers. The expected product size was 746 base pairs. Lanes 1-17 were atherosclerotic plaques 40082-40083, 40088-4009640097-400101.; lanes 20-36 were atherosclerotic plaques 400102-40103, 40105-40108, 40113-40121, 40123-40125; lanes 18 and 37, negative control, no DNA; lanes 19 and 38, positive control, PCR product for *Tanarella forsythiensis* reference strain DNA (ATCC 43037). There was no PCR product for any of the samples.

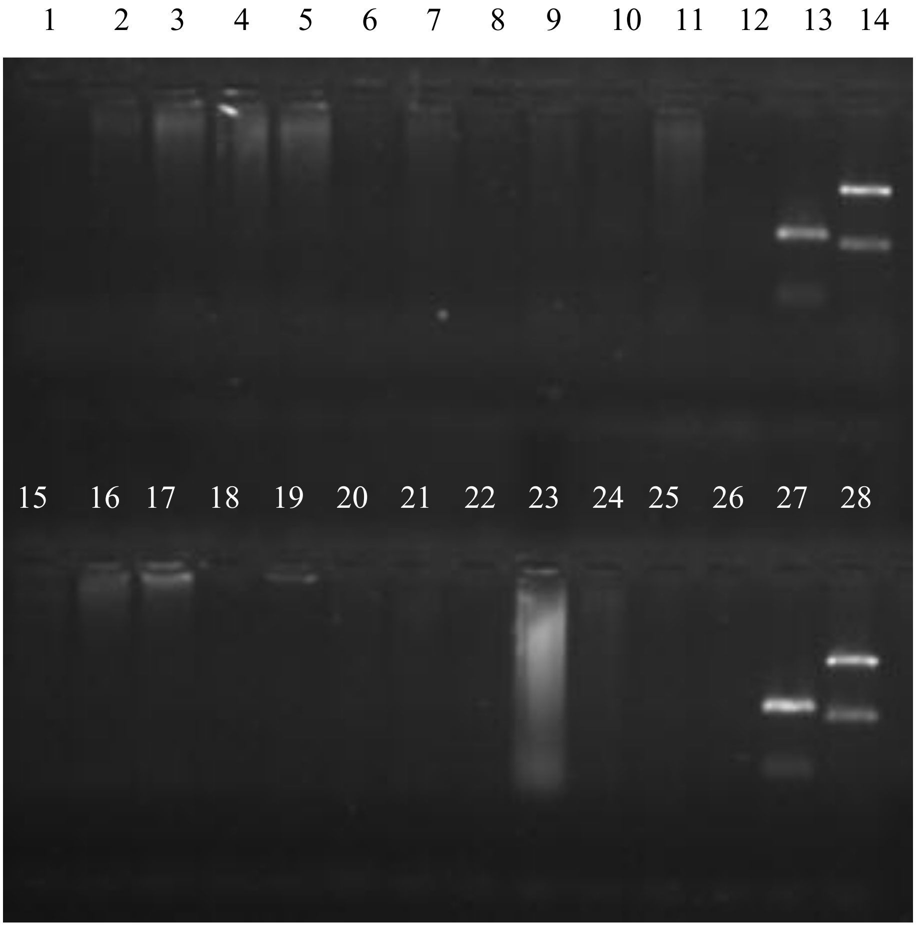

Fig.11. The separation of a PCR product in 1.5% agarose gel for *Treponema denticola* for atherosclerotic plaques or aneurysm vessel walls. 22 atherosclerotic plaques were analyzed. The PCR was conducted with *T.dent*. F and R primers. The expected product size was 316 base pairs. Lanes 1-11 were atherosclerotic plaques 40082-40083, 40088-40096; lanes15-25 were atherosclerotic plaques 40097-40103, 40105-40108; lanes 12 and 26, negative control, no DNA; lanes 13 and 27, positive control, PCR product for *Treponema denticola* reference strain DNA (ATCC 33520). There was no PCR product for any of the samples.

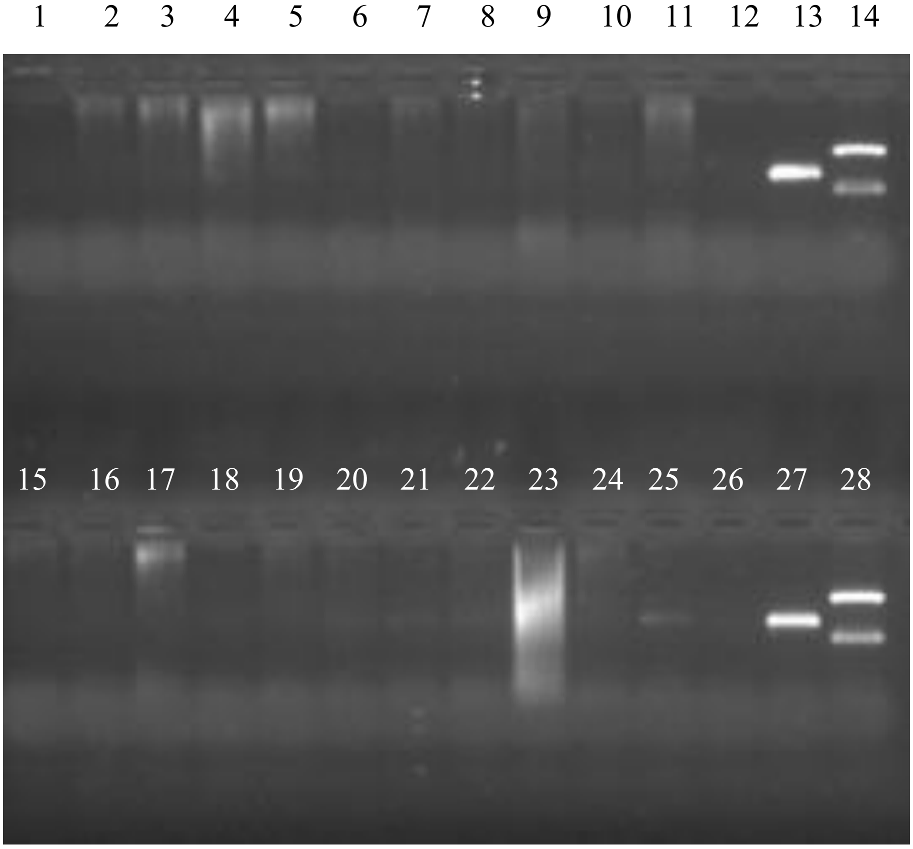

Fig.12. The separation of a PCR product in 1.5% agarose gel for *Prevotella intermedia* for atherosclerotic plaques or aneurysm vessel walls. 22 atherosclerotic plaques were analyzed. The PCR was conducted with *P.int*. F and R primers. The expected product size was 259 base pairs. Lanes 1-11 were atherosclerotic plaques 40082-40083, 40088-40096; lanes 15-25 were atherosclerotic plaques 40097-40103, 40105-40108; tracks 12 and 26, negative control, no DNA; lanes 13 and 27, positive control, PCR product for *Prevotella intermedia* reference strain DNA (15032 ATCC). There was no PCR product for any of the samples.
