# Supplementary material for: Periodontal status and the incidence of selected bacterial pathogens in periodontal pockets and vascular walls in patients with atherosclerosis and abdominal aortic aneurysms
Source: PLoS One. 2022 Aug 11;17(8):e0270177. doi: 10.1371/journal.pone.0270177 (PMC9371326; doi:10.1371/journal.pone.0270177)
Supplement: S1 Raw images — (PDF) [file pone.0270177.s002.pdf]

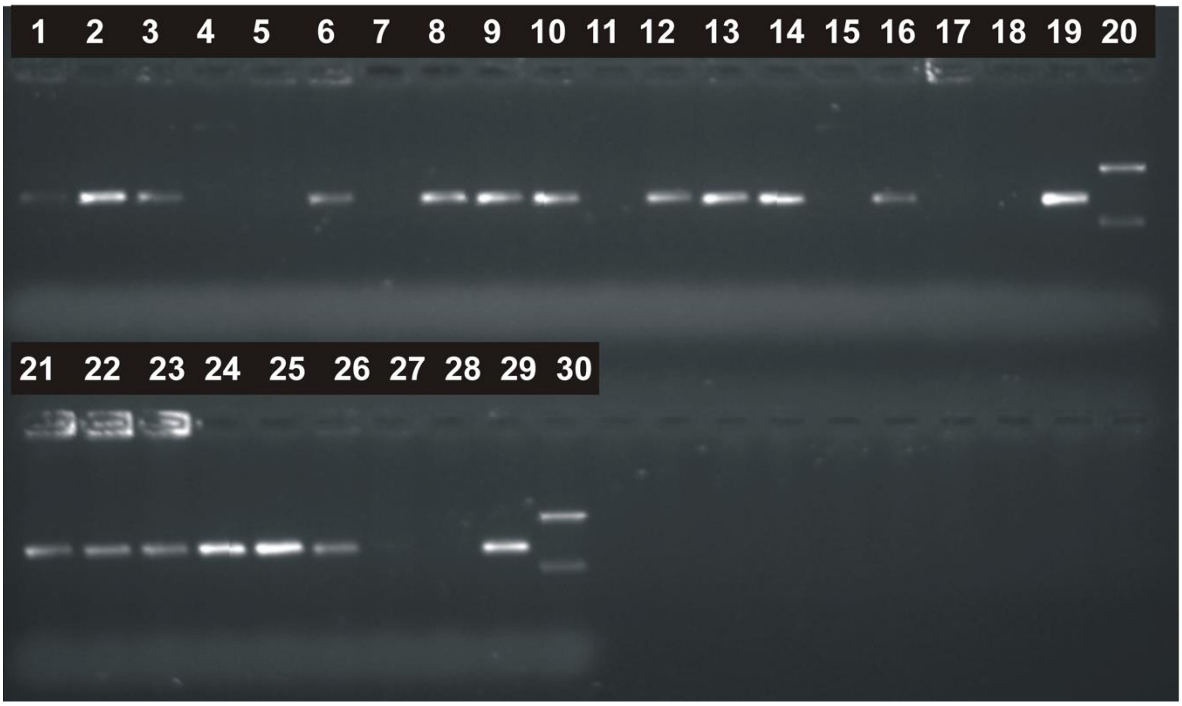

Fig. 7. The separation of a PCR product in 1.5% agarose gel for *Porphyromonas gingivalis* for subgingival plaque samples. 24 subgingival plaque samples were analyzed. The PCR was conducted with *P. gingivalis* F and R primers. The expected product size was 405 base pairs. Lanes 1-17 were subgingival plaque samples 40001-40017; lanes 21-30 were subgingival plaque samples 40018-40024; lanes 18 and 28, negative control, no DNA; lanes 19 and 29, positive control, PCR product (405 bp) for *Porphyromonas gingivalis* reference strain DNA (ATCC 33277D); lanes 20 and 30, size marker, 745 and 267 bp. PCR products were obtained for samples: 40001, 40002, 40003, 40006, 40008, 40009, 40010, 40012, 40013, 40016, 40018, 40019, 40020, 40021, 40022, 40023. However, there were no PCR products for samples 40004, 40005, 40007, 40011, 40017, 40024.

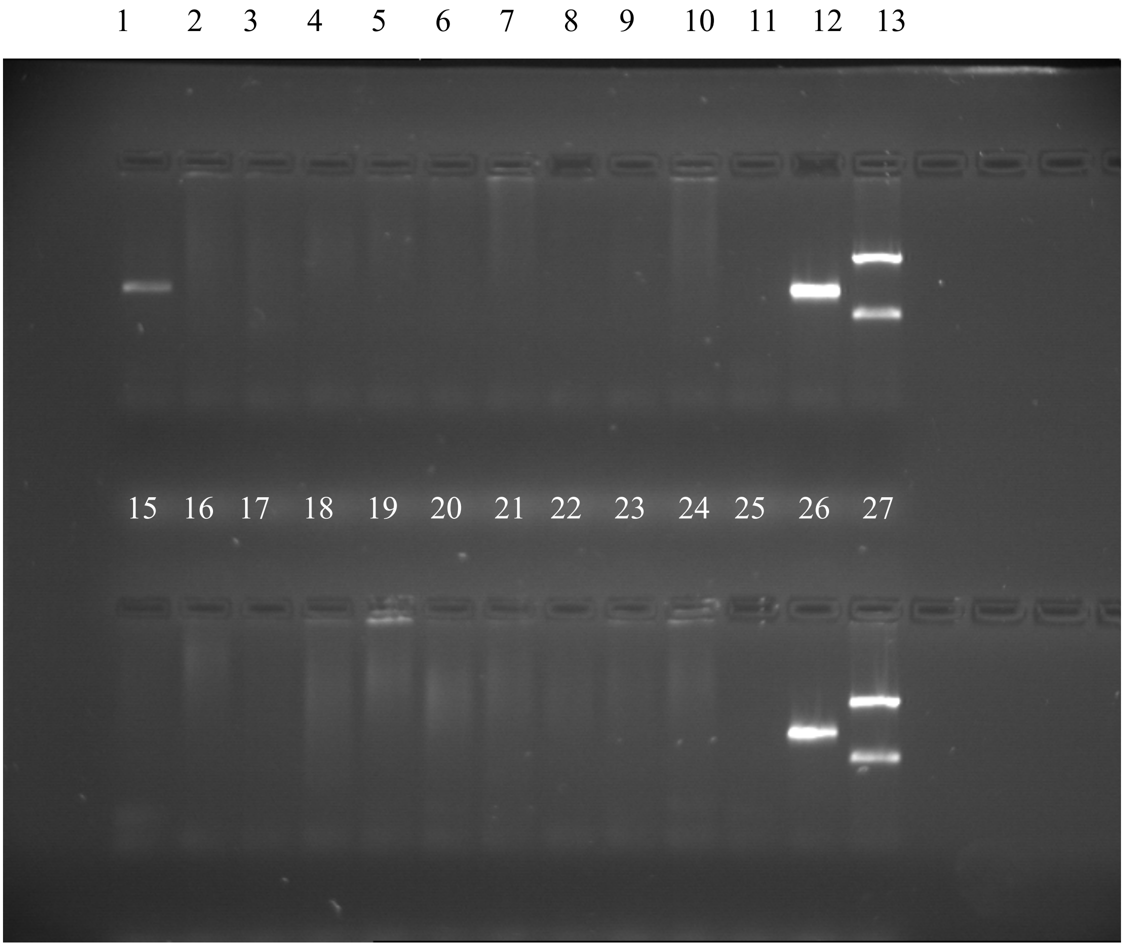

Fig. 8. The separation of a PCR product in 1.5% agarose gel for *Porphyromonas gingivalis* for atherosclerotic plaques or aneurysm vessel walls. A total of 20 atherosclerotic plaques were analyzed. The PCR was conducted with *P. gingivalis* F and R primers. The expected product size was 405 base pairs. Lanes 1-10 were atherosclerotic plaques 40001-40003, 40005, 40010, 40013-40017.; lanes 14-23 were atherosclerotic plaques 40020, 40022-40025, 40031-40032, 40034-40035, 40037; lanes 11 and 24, negative control, no DNA; lanes 12 and 25, positive control, PCR product (405 bp) for *Porphyromonas gingivalis* reference strain DNA (ATCC 33277D), lanes 13 and 26, size marker, 745 and 267 base pairs. PCR product was obtained only for sample 40001.
